# An Ultra-Thin Wearable Thermoelectric Paster Based on Structured Organic Ion Gel Electrolyte

**DOI:** 10.1007/s40820-025-01721-4

**Published:** 2025-03-31

**Authors:** Zhijian Du, La Li, Guozhen Shen

**Affiliations:** School of Integrated Circuits and Electronics, Beijing Institute of Technology, Beijing, 100081 People’s Republic of China

**Keywords:** Flexible thermoelectric devices, Ionic thermoelectric, Organic ion gel, Solvation effect, Thermally rechargeable supercapacitor

## Abstract

Thermoelectric technology that utilizes thermodynamic effects to convert thermal energy into electrical energy has greatly expanded wearable health monitoring, personalized detecting, and communicating applications. Encouragingly, thermoelectric technology assisted by artificial intelligence exerts great development potential in wearable electronic devices that rely on the self-sustainable operation of human body heat. Ionic thermoelectric (i-TE) devices that possess high Seebeck coefficients and a constant and stable electrical output are expected to achieve an effective conversation of thermal energy harvesting. Herein, we developed an i-TE paster for thermal chargeable energy storage, temperature-triggered material recognition, contact/non-contact temperature detection, and photo thermoelectric conversion applications. An all-solid-state organic ionic gel electrolyte (PVDF-HFP-PEO gel) with onion epidermal cells-like structure was sandwiched between two electrodes, which take full advantage of a synergy between the Soret effect and the polymer thermal expansion effect, thus achieving the enhanced ZT value up to 900% compared with the PEO-free electrolyte. The i-TE device delivers a Seebeck coefficient of 28 mV K^−1^, a maximum energy conversion efficiency of 1.3% in performance, and ultra-thin and skin-attachable properties in wearability, which demonstrate the great potential and application prospect of the i-TE paster in self-sustainable wearable electronics.

**Supplementary Information:**

The online version contains supplementary material available at 10.1007/s40820-025-01721-4.

## Highlights

- All-solid-state organic ion gel electrolyte with superior thermal tolerance capability and environmental stability can well penetrate into the electrodes of thermoelectric paster to ensure an excellent interfacial contact.
- Inspired by the “onion epidermal cells” structure, the organic ion gel electrolyte supported by a porous polymer skeleton offers a considerable ZT figure according to the thermal expansion effect.
- Considering the compatibility between electrode and electrolyte, an ultrathin and skin-attachable i-TE paster was assembled to acquire a satisfactory Seebeck coefficient and used in various applications.

**Supplementary Information:**

The online version contains supplementary material available at 10.1007/s40820-025-01721-4.

## Graphical abstract

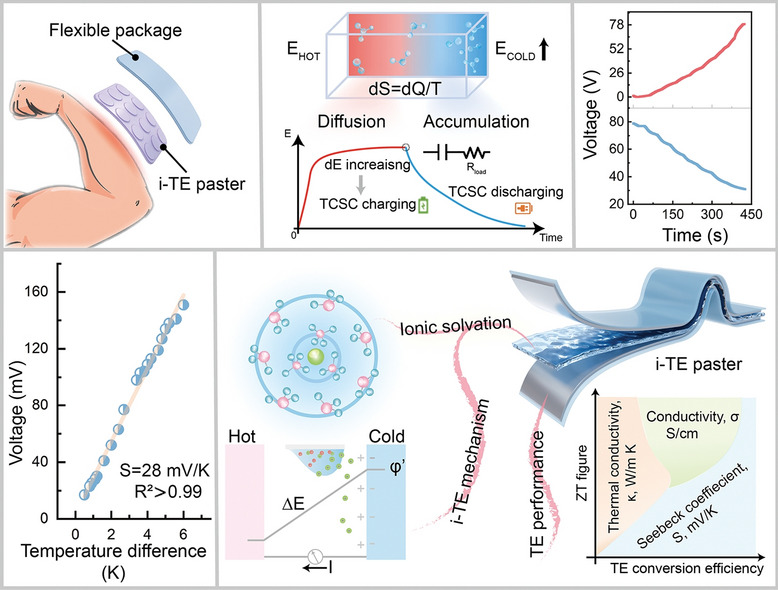

## Introduction

Thermoelectric technology with the design of advanced, flexible, and miniaturized properties has shown a modern and diversified trend as wearable electronic devices, making a big splash in the field of smart healthcare as well as artificial intelligence such as human–computer interaction [1–3]. Different from the previous thermoelectric devices applied in power generation to respond to the persistently harsh global energy landscape, the optimized thermoelectric electronic could provide efficient heat conversion and communication for mechanical devices and human body heat [4, 5]. Great efforts have been devoted to fabricating thermoelectric devices for application in self-driven human temperature acquisition, image recognition, neural simulation, VR/AR interactive haptic sensing, and passive integrated system building [6]. For example, Li et al. proposed a self-powered material identification ring (MIR) based on the thermoelectric effect of the prepared dual-network ionic hydrogel, which can actively infer the type of material without an external power connection by analyzing the voltage signals related to interfacial heat transfer generated in contact with different materials [7]. Luo and co-workers fabricated a self-driven hemispherical retinotopic eye relying on the photothermoelectric effect of photosensitizer-ionic gel heterojunction, which converts incident photons into neuroelectrical signals with retina-like tunable plasticity, achieving the restoration of intrinsic perceptual functions [8].

High-efficient thermoelectric devices require specific materials to directly convert heat and electric energy into each other. Traditional electron-type thermoelectric materials based on the Seebeck effect are mostly concentrated in materials with high conductivity, carrier mobility, and outstanding energy filtering effects, including inorganic semiconductors composed of alloys, ceramics, and metal oxides, and conductive polymers, such as polyaniline and polythiophene, which suffers very low thermal power less than 10 µV K^−1^ [9, 10]. As a result, ionic thermoelectric (i-TE) materials with ions as carriers are developed, which rely on two kinds of REDOX effect and Soret effect to realize the collection of low-grade heat (LGH), and especially enrich the thermal energy management system [11–13]. Hydrogel electrolyte as a highly hydrophilic three-dimensional mesh material is widely used in the fabrication of a variety of solid-state i-TE devices [14]. However, the poor dehydration and thermal tolerance capability of the hydrogel makes it difficult to apply in long-life and stable i-TE devices [15, 16]. Therefore, developing an i-TE device based on solid-state gel electrolyte with high heat conversion efficiency and superior thermal tolerance capability is extremely urgent for self-sustainable wearable electronics.

In this work, we developed an organic gel-based i-TE paster to address the irreversible variability in the performance output of the device due to environmental effects and greatly enhance the robustness of the device. Inspired by the “onion epidermal cells” structure, the organic ion gel electrolyte supported by a porous polymer skeleton offers a considerable ZT figure according to the thermal expansion effect. The assembled i-TE paster can output a stable Seebeck coefficient of 28 mV K^−1^, which supports the i-TE paster in the application of thermally chargeable energy storage, temperature-triggered material identification, contact/non-contact temperature detection, and opto-thermoelectricity.

## Experimental Section

### Material Synthesis

#### Materials

Acetone (> 99%), dimethyl carbonate (DMC, 99.9%, water ≤ 50 ppm), ethylene carbonate (99%), lithium trifluoromethanesulfonate (LiTf, 98%), poly(vinylidene fluoride-co-hexafluoropropylene) (PVDF-HFP, average Mw ~ 455,000, average Mn ~ 110,000), and polyethylene oxide (PEO, average Mv ~ 5,000,000) were provided by Innochem. Poly(3,4-ethylenedioxythiophene (PEDOT, 1.9%) was purchased by OE Chemicals Co., Ltd. Polyaniline (PANI, 2.5%) was purchased by Ninghua Dongxi Chemical Co., Ltd. All chemicals were used directly without further purification. Deionized water (DI water) was used throughout the experiments. Ti_3_C_2_T_*x*_ MXene was obtained by selective etching Ti_3_AlC_2_ MAX, which was carried out according to our previous work [17].

#### Preparation of the Ion Organic Gel Electrolyte

The solution casting method was adopted to prepare the electrolyte membrane. 0.04 g PVDF-HFP and 0.002 g PEO dissolved in 15 mL acetone to obtain the precursor fluid. The solution was poured into a custom-made groove made of quartz glass. And then, just wait for the precursor fluid to evaporate naturally at room temperature. The volatilized translucent membrane continued to dry overnight at a vacuum, 80 °C to obtain a white membrane with the microstructural. The gel was obtained by the sol–gel method. The gelating solvent was prepared by mixing DMC, EC, and LiTf in proportion. Then put the prepared membrane into the gelating solvent and soak for more than an hour to obtain a transparent organic gel.

#### Preparation of PANI-C@PI Electrode Film

Structured Carbon material was sintered on a 100 µm PI film with the help of a CO_2_ laser cutting machine. Then PANI solution was sprayed on the structured carbon material and waited for the surface moisture volatilization at room temperature to acquire PANI-C@PI electrode film. Substitutively, PEDOT and Ti_3_C_2_T_*x*_ MXene aqueous solutions were sprayed on to obtain PEDOT-C@PI and MXene-C@PI films, respectively.

### Characterization

X-ray diffraction (XRD) patterns were collected on Rigaku D/Max-2550. The elements and bonding were analyzed by X-ray photoelectron spectroscopy (XPS, Thermo ESCALAB 250Xi). Scanning electron microscope (SEM) images were collected using NANOSEM 650-6700F. The surface potential was tested by AFM (Bruker, Dimension Icon), and the vibration state of the material was tested by Raman (Renishaw inVia). Contact Angle measurement was done by the contact Angle measuring instrument (Dataphysics OCA50). ZETA potential was obtained by Nanoparticle size and Zeta potential analyzer (Nano ZS90, Malvern). The absorbance of the material was acquired by UV–VIS-NIR (UV3600, Shimadzu). In situ infrared spectroscopy was done by FTIR spectrometer (Nicolet iS50, ThermoFisher Scientific). The dielectric constant of the material was obtained by the impedance analyzer (Agilent 4294A). All electrochemical tests were performed at room temperature using the CHI-760D Electrochemical workstation.

## Results and Discussion

### Wearable i-TE Paster Enables the Response and Collection of Human Body Heat

**Fig. 1:**
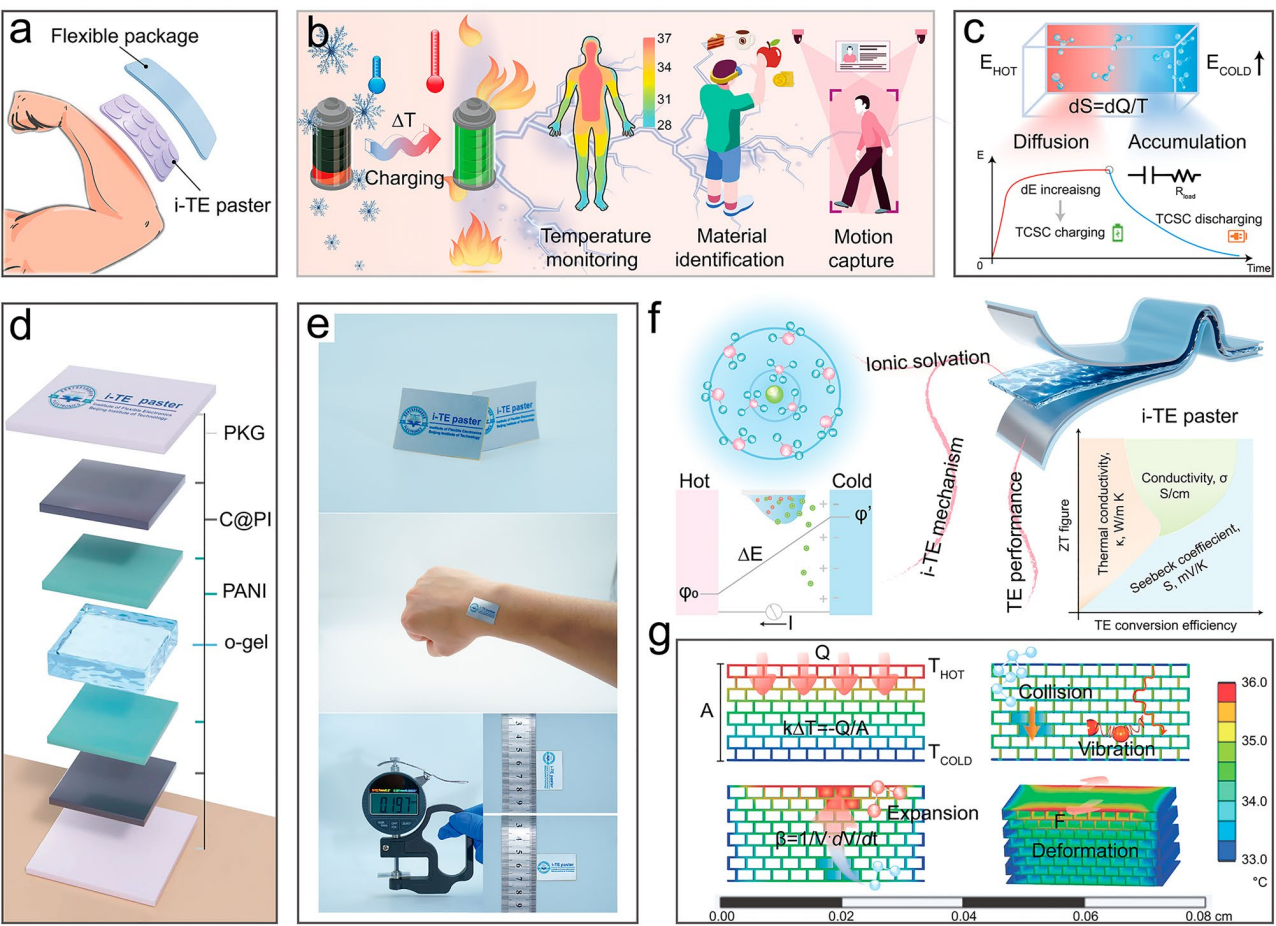
Wearable i-TE paster enables the response and collection of heat. **a** Conceptual graph of the flexible i-TE paster that can be attached closely to the skin. **b** Schematic graph of the i-TE paster that can realize temperature monitoring, material identification, and motion capture. **c** Working mechanism of the thermal chargeable supercapacitor (TCSC) based on ionic thermoelectric effect. **d** Structure diagram of the assembled i-TE paster. **e** Optical photographs of the i-TE paster. **f** Schematic diagram of the ionic solvation, i-TE mechanism, and TE performance relating to the i-TE paster. **g** FEA simulations for the polymer electrolyte film according to the heat transfer effect and thermal strain phenomenon

Generally, the human body is considered to be an eternal heating body because the body needs heat for the exchange and signaling of substances within cells, the operation of the immune system, and the work of various organs and tissues [18]. However, most of the heat gained metabolically from ingesting food is absorbed by the environment as low-grade waste heat [19]. Therefore, based on the Soret effect and electric double layer (EDL) theory, the original thermal chargeable supercapacitors (TCSCs) have been developed by combining the ionic thermoelectricity (i-TE) and supercapacitor, which enable to conversion of the heat emitted from the human skin into electricity, and at the same time store electrical energy, when the device is tightly attached to the skin (Fig. 1a) [20]. Moreover, as shown in Fig. 1b, i-TE technology has been also used to acquire temperature information due to the ultra-high sensitivity compared to electronic thermoelectricity (e-TE). In addition, thanks to i-TE, the precise identification of different materials and motion capture have been achieved depending on the inherent thermal conductivity and photo thermoelectric effect. The thermal movement of ions induces chaos at the high-temperature zone, which enables the entropy to increase in the system. To achieve equilibrium renewedly, ions will actively migrate to the cryogenic zone and aggregate, resulting in a changed potential, and thus generate an electromotive force (EMF) between the two ends of the system [21]. Sequentially, the i-TE device will be manufactured by designing a special electrode that can adsorb the charged ions and induce the production of opposite charges internally due to the electrostatic forcing (Fig. 1c). In practice, as shown in Fig. 1d, a flexible and wearable i-TE paster was assembled with a symmetrical structure, including the organic gel electrolyte film (o-gel), polyaniline films (PANI), carbonized PI films (C@PI) and package films (PKG). Figure 1e shows some optical photographs of the i-TE paster and its mechanical stability when wearing. After measuring, its length and width are 3 and 2 cm, respectively. And its thickness is only 0.197 mm. Figures S1 and S2 illustrate the appropriate breathability and oxygen permeability of the encapsulation material employed in the i-TE paster, meeting the medical wear standards, which suggests a robust device-skin interface for long-term stable operation as well as good wearing comfort. Additionally, we proceeded to stick i-TE pasters on the back and abdomen of the mice. Then, the histopathological sections of the involved tissues were performed 7 days later. The H&E stained histopathologic images of the outer layer of the epidermis are shown in Fig. S3. There were no obvious abnormalities such as erosion, inflammation, or edema at either site, confirming the bio-friendly, medical-grade encapsulation strategy. As pictured in Fig. 1f, a “sandwich” structure was adopted to prepare the i-TE paster, consisting of an electrolyte layer and two upper and lower electrode layers. On this basis, we discussed in detail the effect of ionic solvation in the gel electrolyte on the output of excellent thermoelectric behavior of the material, and a sufficient study of the mechanisms was conducted involved in the device. Furthermore, an “onion epidermal cells” structure supported polymer film was designed to offer the electrolyte skeleton, and Fig. 1g demonstrates the advantages of choosing this custom structure from the perspective of heat transfer and thermal strain via FEA simulations (Discuss in detail the statement in Note S1).

### Design of the Organic Electrolyte Film Based on "Onion Epidermal Cells" Structure

**Fig. 2:**
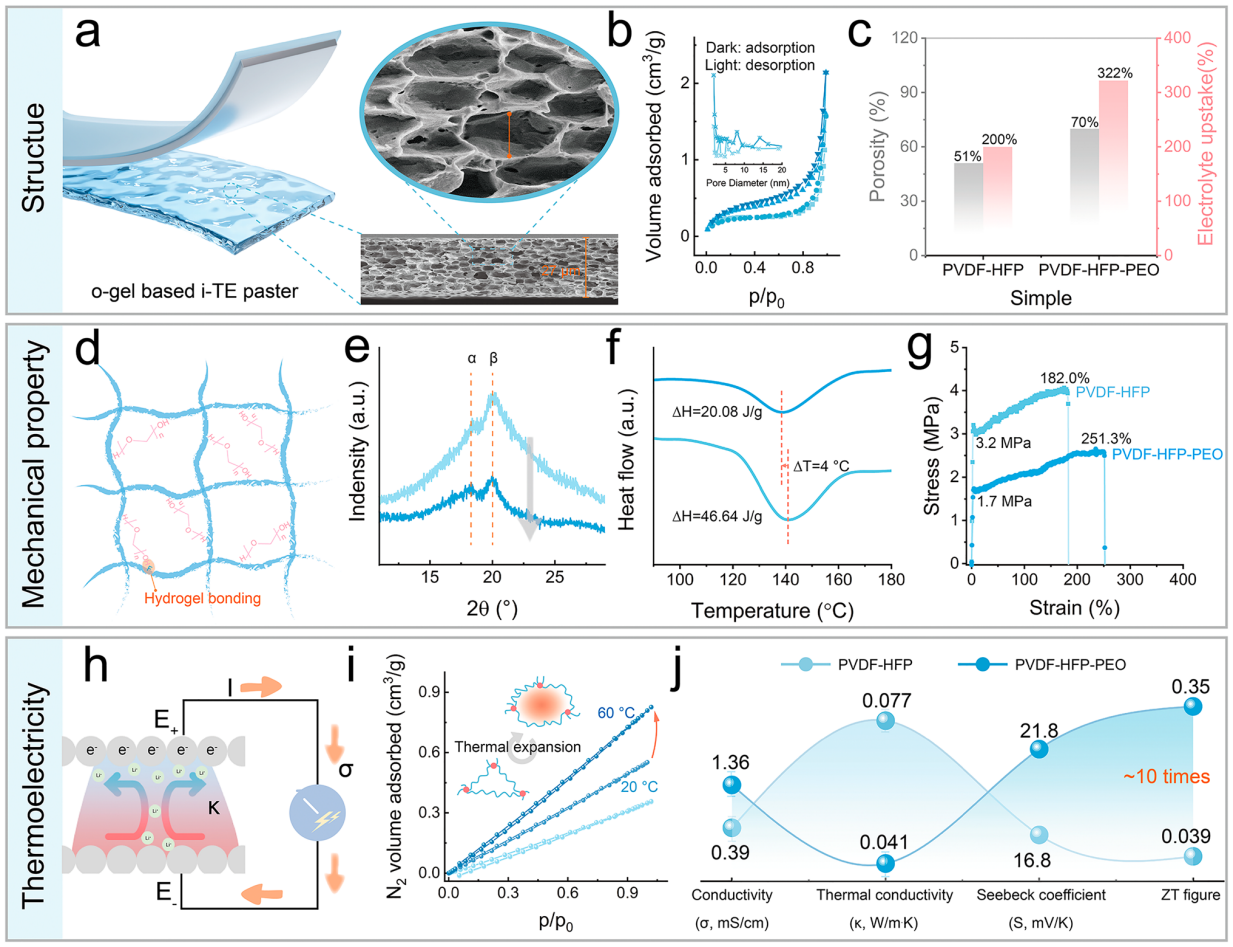
Comparison of PVDF-HFP and PVDF-HFP-PEO on structure, mechanics, and electricity. **a** Sectional SEM images of PVDF-HFP-PEO membranes. **b** N_2_ adsorption curves of PVDF-HFP and PVDF-HFP-PEO membranes. **c** Porosities and electrolyte uptakes of PVDF-HFP and PVDF-HFP-PEO membranes. **d** Diagram of hydrogen bonding between PVDF-HFP and PEO. **e** XRD patterns of PVDF-HFP and PVDF-HFP-PEO membranes. **f** DSC curves of PVDF-HFP and PVDF-HFP-PEO membranes. **g** Stress–strain curves of PVDF-HFP and PVDF-HFP-PEO membranes. **h** Power generation mechanism diagram of i-TE. **i** N_2_ adsorption curves at 20/60 °C of PVDF-HFP and PVDF-HFP-PEO membranes. **j** Electrical conductivity, thermal conductivity, Seebeck coefficient, and ZT figure of PVDF-HFP and PVDF-HFP-PEO gels. (PVDF-HFP is indicated as light blue, PVDF-HFP-PEO is indicated as dark blue)

Figure 2a shows a tightly stacked hollow polyhedral structure in the prepared electrolyte gel, like the onion epidermal cells, which originates from the crystallization of PVDF-HFP at room temperature and then pore expansion occurring in a high temperature and vacuum environment. As shown in Fig. S4, the surface of the membrane presents a hollow-out morphology, which in particular increases the contact area between the electrolyte and the electrode. Compared with PVDF-HFP, the composite membrane after doping PEO presents denser and uniform in SEM images (Fig. S5). In addition, Fig. 2b displays the PVDF-HFP-PEO membrane with a stronger N_2_ volume absorption, which proves some pores with larger sizes were prepared under the action of PEO chains. Combined with the porosities of the membranes obtained via the n-butanol calibration method, PVDF-HFP-PEO copolymer shows more outstanding electrolyte uptakes and the results are listed in Fig. 2c. As shown in Fig. 2d, due to the intermolecular hydrogen bonding effect between PVDF-HFP and PEO, the polymer chains in the copolymer present more disordered, which contributes to the formation of a low-crystalline 3D polymer network. Sequentially, the intensities of α and β peaks from PVDF-HFP, shown in XRD patterns (Fig. 2e), are both crippled; and then a lower phase-transition temperature and a smaller enthalpy change (∆H) of the copolymer are acquired and displayed in DSC curves (Fig. 2f). On the one hand, a weakened crystallinity induced a more flexible membrane, and Fig. 2g shows PVDF-HFP-PEO membrane has lower yield stress (1.7 MPa) and a considerable percentage of breaking elongation (251.3%). On the other hand, the heat transfer process from the thick-oriented is limited due to the thin crystallinity, and thus it can be assumed that the copolymer has a lower thermal conductivity. As shown in Fig. 2h, the generation mechanism of ionic thermoelectricity can be summarized as the directional diffusion of ions in the electrolyte under the action of temperature difference, resulting in a potential difference between the two electrodes, so that the cold side and the hot side become positive and negative, respectively, and the current will pass from the positive to the negative through the external circuit [22]. Based on the charged property of the moving ion, i-TE materials that active ions carry positive charges are defined as p-type, otherwise are defined as n-type. Figure 2i proves the phenomenon of pore thermal expansion that occurs in porous PVDF-HFP-PEO membranes at high temperatures, which is attributed to the self-shrinking/-expanding properties of thermosensitive PEO polymer chains at low/high temperatures [23]. Therefore, PEO-doped copolymers can obtain a satisfactory thermoelectric performance because carriers can diffuse more actively in the expanding pore (Figs. S6 and S7). The properties of thermoelectric materials are evaluated by the figure of merit ZT, which is further expressed by the Seebeck coefficient (S), electrical conductivity (σ), and thermal conductivity (κ) (ZT = S^2^σT/κ). It is worth mentioning that all the thermoelectric behavior tests were carried out on a homemade cold/heat supply platform, and its planning diagram and mechanism are described in Note S2. Figure 2j lists some parameters, and the related data are shown in Figs. S8 and S9. First, PVDF-HFP-PEO after gelation has a larger electrical conductivity, which benefits from more electrolyte uptake. Secondly, the low thermal conductivity of the copolymer gel confirms the weakened crystallinity of PVDF-HFP. Thirdly, PVDF-HFP-PEO gel has a more prominent Seebeck coefficient, which is the result of synergy between the abundant carrier content and polymer skeleton that can undergo thermal phase transition. Finally, a satisfactory ZT figure of PVDF-HFP-PEO gel is calculated and nearly ten times larger than that of PVDF-HFP gel, which demonstrates the doping of PEO especially promotes the output of thermoelectric behavior of PVDF-HFP-based copolymer gel. In particular, its ZT value (0.35) reaches the standard of traditional inorganic e-TE materials (0.4), and some comparisons are also listed in Table S1 [24]. Besides, the room temperature dielectric spectrum on PVDF-HFP and its derivative was further carried out to characterize the electrical properties, and the detailed description and data are shown in Note S3.

### Effect of the Organic Electrolyte System on i-TE Performance

**Fig. 3:**
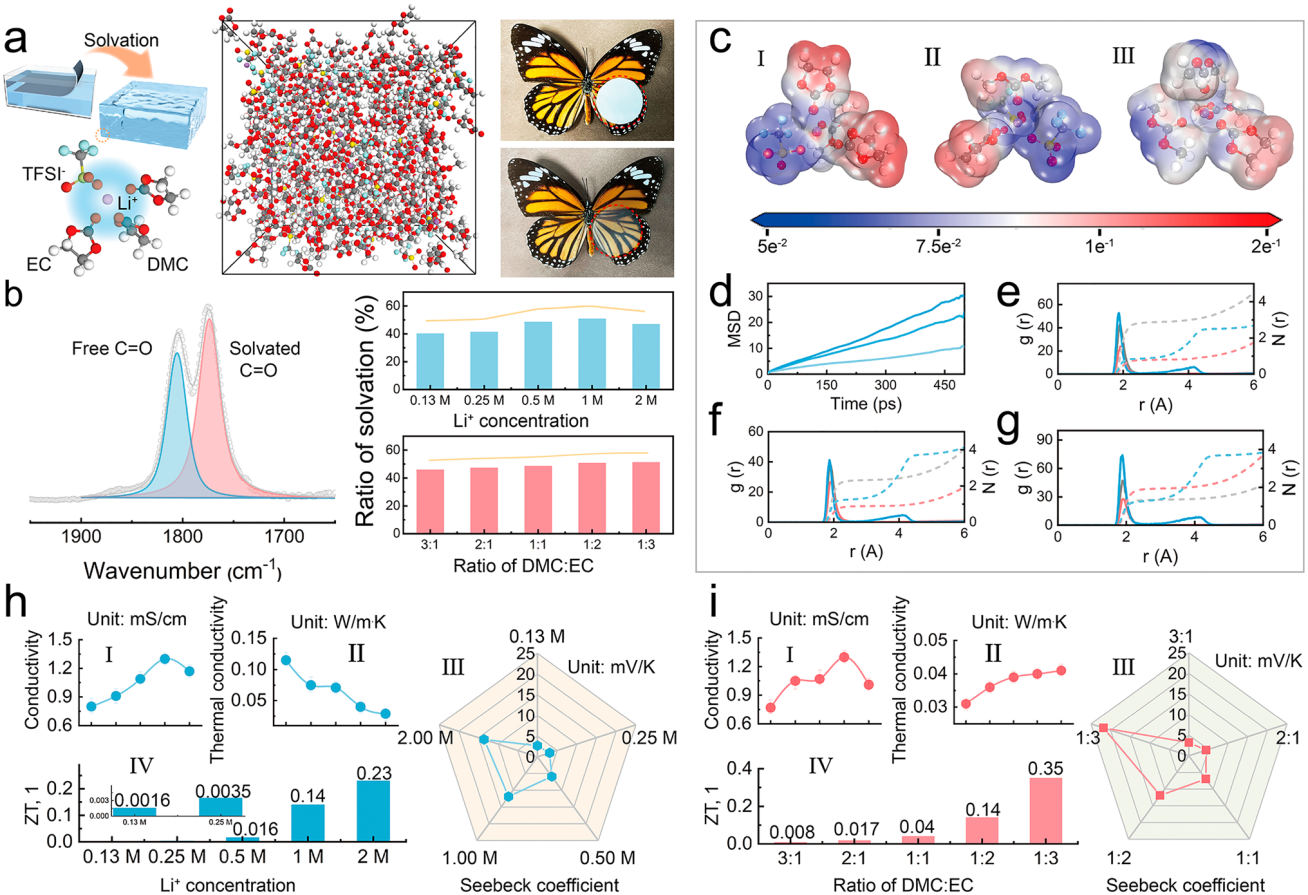
Effects of the Li^+^ concentrations and the ratios of solvent DMC: EC on electrical properties. **a** Schematic diagram of the solvation structure and optical photographs of the PVDF-HFP-PEO membrane before and after gelation. **b** Intensity of solvation of the different Li^+^ concentrations and the different ratios of DMC: EC. **c** Molecular electrostatic potential (ESP) maps of 1 M LiTf DMC: EC = 1:2 (I), 1 M LiTf DMC: EC = 2:1 (II), 2 M LiTf DMC: EC = 1:2 (III). **d** Mean-squared displacement (MSD) of 1 M LiTf DMC: EC = 1:2 (dark blue), 1 M LiTf DMC: EC = 2:1 (blue), 2 M LiTf DMC: EC = 1:2 (light blue). **e ~ g** The radial distribution function (RDF) of 1 M LiTf DMC: EC = 1:2 (**e**), 2 M LiTf DMC: EC = 1:2 (**f**), 1 M LiTf DMC: EC = 2:1 (**g**). **h** Conductivity (I), thermal conductivity (II), Seebeck coefficient (III), and ZT figure (IV) of the different Li^+^ concentrations. **i** Conductivity (I), thermal conductivity (II), Seebeck coefficient (III), and ZT figure (IV) of the different ratios of DMC: EC

The PVDF-HFP-PEO copolymer film prepared by solution casting method, as the polymer framework, was gelatinized in immersion into a configured electrolyte solution, where the electrolyte was a mixed carbonate-based solution (DMC and EC). As shown in Figs. 3a and S10, it can be found that the gelatinized polymer film changes from white to transparent and the yield point from the stress–strain curve disappears, which means the formation of gels. The organic gel is considered to be a quasi-solid substance, and the electrolyte solution involved in gelation is identified as an independent monomer, which is the main factor affecting its electrical performance output [25, 26]. Therefore, active attention to the ionic solvation occurring in the electrolyte is necessary to achieve high electrical performances. For the mechanism of ionic thermoelectricity generation, the highly polar solvent can promote the dissociation of LiTf, and the high concentration of Li^+^ ion obtained in the system, as a carrier, induces a strong electric double layer (EDL) effect on the electrode, thus generating a satisfactory thermoelectric performance [27]. However, the solvent molecules are wrapped around the Li^+^ ions, forming ion clusters that inhibit the directional migration of ions under an electric field [28]. In the system, the concentration of LiTf salt and the ratio of carbonate solvent are considered the main reasons for determining ionic solvation. Figure 3b shows the intensities of ionic solvation (IS) that occurs in various components characterized by FT-IR (absorption wavenumer = 1775 cm^−1^ regarded as solvated C = O; absorption wavenumer = 1803 cm^−1^ regarded as free C = O; IS value regards as the ratio of solvated C = O and free C = O) [29]. On the one hand, IS presents the maximum value in the electrolyte dissolving 1 M LiTf, while more salts tend to inhibit the solvation of Li^+^ ion due to incomplete dissolution. On the other hand, the involvement of more highly polar EC molecules leads to a more significant increase in IS values, which can be attributed to adsorption from oxygen-containing functional groups. Then, as shown in Figs. 3c ~ g, according to molecular dynamics, a series of simulations and calculations were carried out to illustrate the phenomenon, which the detailed demonstration and explanation are described in Note S4. Subsequently, the electrical conductivities, thermal conductivities, Seebeck coefficients, and ZT figures were orderly acquired via a series of tests. Firstly, as shown in Fig. 3h I, similar to the results of IS, the electrolyte with 1 M LiTf dissolved outputs the maximum electrical conductivity, which is the synergistic effect of ion concentration and ion mobility barriers (E_a_). On the one hand, the more prominent solvation leads to a more abundant concentration of Li^+^ in the fraction solvated by the 1 M salt; on the other hand, the realistic lower E_a_ in the Arrhenius fitting diagram elucidates that these ions can undergo smooth migration in response to the electric field (Fig. S11). Figure 3h II shows the thermal conductivities of the different LiTf concentrations, where the viscosity of the solution plays a decisive role directly (Fig. S12). The increase in salt concentration caused a gradual increase in the Seebeck coefficient throughout the tested range, which indicates that the multi-ion complexes formed by high concentrations of lithium salts are also involved in the thermal diffusion process based on the effect of temperature difference and output larger thermal voltages (Fig. 3h III). Moreover, ZT figures also show an increasing trend, which is the result of the combined action of three physical parameters due to the formula of the ZT value (Fig. 3h IV). Secondly, as shown in Figs. 3i I and S13, the high viscosity of EC makes the mixed electrolyte viscous, and by affecting the fluidity of the electrolyte, the migration of Li^+^ ion is inhibited, and the E_a_ becomes larger (Fig. S14). Therefore, the electrical conductivity of the gel electrolyte decreases when DMC: EC = 1:3. More EC molecular with the nature of strong vibration enable the heat conduction and thus lead to an increase in thermal conductivity (Fig. 3i II). Subsequently, the high polarity of the EC molecule also leads to an increase in IS value. With the increasing ratio of DMC and EC in the electrolyte, there are more free ions undergoing rapid ion migration. Therefore, as shown in Figs. 3i III and IV, the Seebeck coefficient and ZT tend to get bigger. In summary, the excellent thermoelectric performance of the electrolyte output is determined by the ionic concentration and polarity of the solvent in the system. In addition, the doping of EC with a high dielectric constant promotes the mixed electrolyte with a higher melting point to maintain liquid stability in the environment, and then the interaction force between the mixed electrolyte with increased polarity and the PVDF-HFP framework becomes more pronounced, which to a large extent also enhances the stabilizing presence of the electrolyte in the gel, and reduces the likelihood of the occurrence of leakage that results in deterioration of the performance [30]. Figure S15 shows the negligible mass change of PVDF-HFP-PEO gel in a constant environment of 50 °C to further evaluate the satisfactory thermostability.

### Promoting the Thermoelectric Performance of i-TE Paster by Electrode Selection

**Fig. 4:**
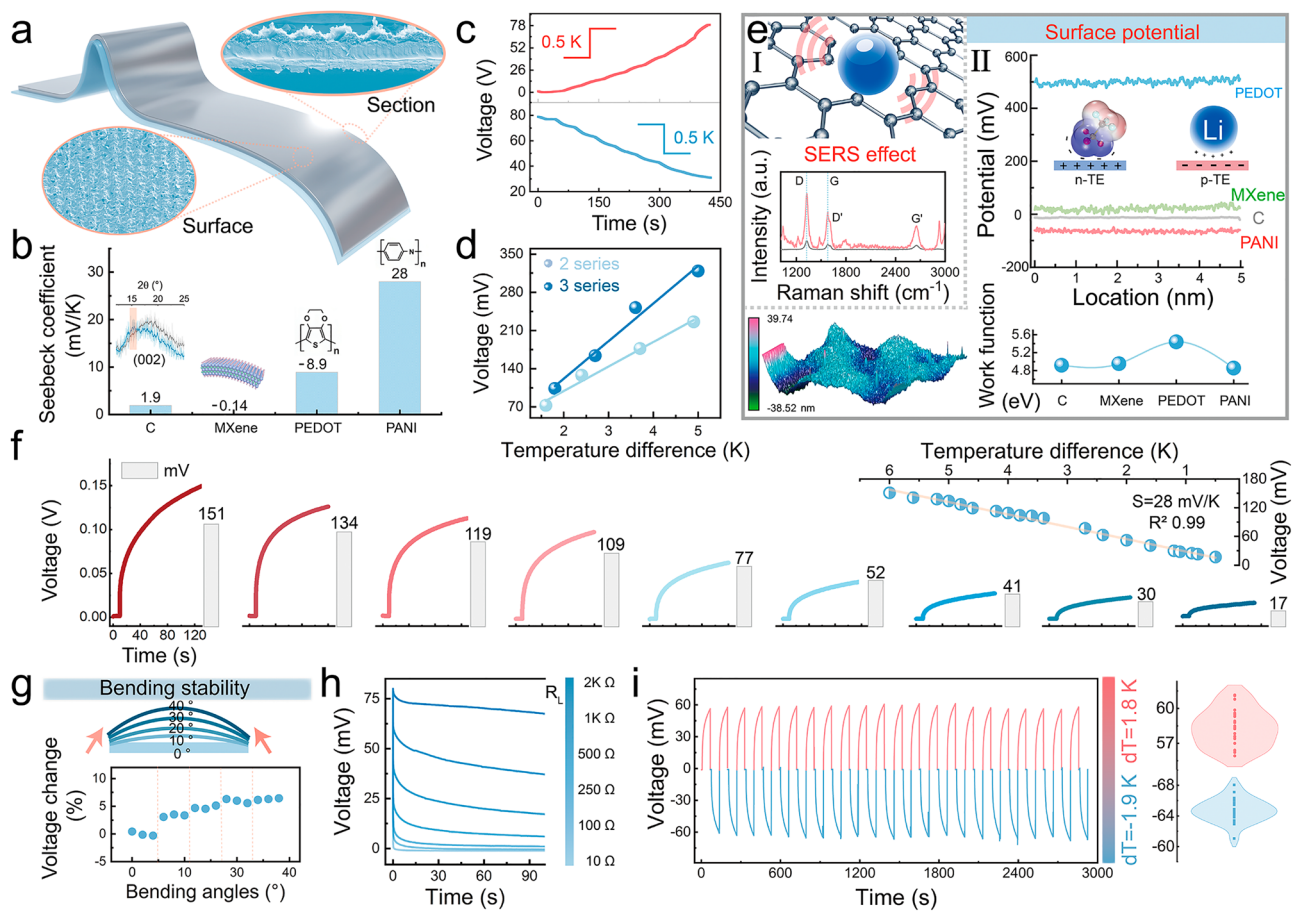
The output thermoelectric performance of the i-TE device assembled with the flexible PANI-C@PI electrode film. **a** Surface and section SEM images of the PANI-C@PI film. **b** Seebeck coefficients of the device assembled with C@PI, MXene-C@PI, PEDOT-C@PI, and PANI-C@PI, respectively. **c** Output voltage curves of the i-TE device in the process of gradually increasing and decreasing the applied temperature difference. **d** Thermal voltages generated and Seebeck coefficients calculated under different temperature differences when two and three i-TE components are connected in series. **e** Characterization of electrode properties: (I) Raman spectra of PANI-C@PI before and after soaking in the electrolyte; (II) the surface potentials and work functions of the four electrodes. **f** Thermal voltage generated by the device assembles with PANI-C@PI under different temperature differences. **g** Output voltage changes at ΔT = 2 K under applying the different bending angles to the device. **h** Discharge voltage curves of the device during discharging when connected to different external loads. **i** Multi-cycle charge and discharge of the device under ΔT = 1.8 and -1.9 K

The CO_2_ laser sintering method was employed to obtain a novel electrode film with high performance and softness, which was further assembled with the prepared organic gel in a sandwich structure to manufacture a wearable i-TE paster. A pyramid-like convex structure appeared on the PI film after laser sintering, as shown in Figs. 4a and S16, these pyramids are constructed by 1D carbon fibers stacked and crossed to achieve fast electronic conduction. The Raman spectrum shown in Fig. S17 proves the sintered carbon on the PI film is graphene-like and, according to its XRD pattern, the structure can be identified as the porous foamy structure with high defects. To solve the problem of morphological instability of foamy carbon under applied stress, some materials, that are easy to form film such as Ti_3_C_2_T_*x*_ MXene, PEDOT, and PANI, were covered with carbon by spraying (Fig. S18). After a series of bending stresses are applied to the film, the conductivity decreases ignorably with the increase of the bending angles, which proves the stability of the electrode. (Fig. S19). Then, a series of i-TE devices were assembled with the different prepared electrode films and their thermoelectricity performance was carried out. As shown in Fig. 4b, c, the i-TE device assembled with PANI-C@PI film can acquire the most outstanding positive Seebeck coefficient of 28 mV K^−1^, and its voltage output presents an obvious ladder upward trend during heating and cooling at intervals of about 0.5 K temperature difference (Fig. S20). In addition, when some components were connected in series, the multiplied Seebeck coefficient was tested out, and a 71.2 mV K^−1^ can be even obtained under the connected three components (Fig. 4d). Figure 4e continues to make some discussion on the different Seebeck coefficients obtained by the i-TE devices assembled with the four electrodes, respectively. In the beginning, the carbonated PI film has been demonstrated to have the ability to support the electrolyte output thermal voltage as an electrode, which can be attributed to the porous graphene structures with high-density defect sites. Figure S21 shows the generation of a large number of oxygen-containing end groups outside the surface of graphene defects, which have been proven to enable selective adsorbing metal ions. Combined with C *sp*^3^ growing at the defect site and C *sp*^2^ with a strong electron delocalization effect, Li^+^ ions diffused from the hot end of the electrolyte can induce charge rearrangement on the electrode surface [31]. As shown in Fig. 4e I, the surface-enhanced Raman scattering (SERS) effect, which occurred after the C@PI was soaked with electrolyte, further reflects the mechanism that ions induce potential changes on the electrode surface to generate electromotive force [32]. In addition, as shown in Figs. S22 and S23, the introduction of Ti_3_C_2_T_*x*_ MXene on the carbonized PI surface also acquires the negligible SERS effect, which confirms a low negative Seebeck coefficient of the i-TE device assembled with MXene-C@PI electrode, considering the small positive surface potential and the larger contact angles to the electrolyte. Moreover, the large positive surface potential from PEDOT-C@PI confirms the device with a Seebeck coefficient of -8.9 mV K^−1^, which is derived from the electrode's adsorption of anions in the electrolyte, and enables the n-TE device (Fig. 4e II). Subsequently, an outstanding Seebeck coefficient obtained by the device assembled with PANI-C@PI electrode can be explained for the following reasons: (1) in Fig. S24, PANI can be proved to promote the adsorbed intensity of Li^+^ ions by increasing the vibrational strength of C *sp*^3^, C *sp*^2^ and C-O bonds, based on the resonance effect (the obvious red shift); (2) the introduced C-N and C = N bonds enable the increase of adsorption sites in the electrode; (3) a considerable negative surface potential of PANI-C@PI can induce the generation of the significant change in potential after ion adsorption; (4) as shown in Fig. S25, the undulating mountain structure displayed on the surface of PANI-C@PI film can increase the contact area between the electrode and the electrolyte, which induces more ions to change the potential of electrode. In addition, the PANI@C-PI film shows a smaller work function, which demonstrates that the outflow of electrons inside the electrode just needs to overcome a lower barrier after ions are adsorbed on the surface of the electrode. And, the Seebeck coefficients of some devices published in other works referring to ionic thermoelectricity are listed in Table S2 and compared with that of our device.

As shown in Fig. 4f, the gradient thermal voltages were acquired, which can verify the strong temperature difference following the behavior of the device, and there is a clear linear relationship for the output voltage at the applied 20 temperature difference points. Then, a series of the output currents continued to be tested out and shown in Fig. S26, which likewise reflects the strong temperature difference following behavior. In addition, under 0 ~ 40° extrusion angles applied, the fluctuations in the output voltage can be negligible, demonstrating the thermoelectric stability of the device (Fig. 4g). Then, a series of the discharge voltage curves after connecting to different external loads illustrate the device can generate a stable output due to the voltage slowly decreasing to a nonzero constant value all the time (Fig. 4h). Subsequently, some opposite voltages output by the device are shown in Fig. S27, when the cold/hot source applied to the two ends of the device is switched, and several temperature differences are applied. The results reflect its reproducibility can reach over 96.6%. Furthermore, we continued to test the multi-cycle repeated thermal charge–discharge behaviors of the device, when applying both forward and reverse temperature differences, respectively, to emphasize the satisfactory thermoelectric stability. It can be seen from Fig. 4i that when ΔT = 1.8 K, the fluctuation of the output thermal voltage is kept within 3% during 22 cycles, and at ΔT = -1.9 K, the fluctuation also remained within 5%, which proves the devise’s real potential in the thermoelectric conversion of intermittent heat sources.

### Applications of the i-TE Paster in the Field of TCSC, Material Identification, Contact/Non-Contact Thermal Response, and Photo Thermoelectricity

**Fig. 5:**
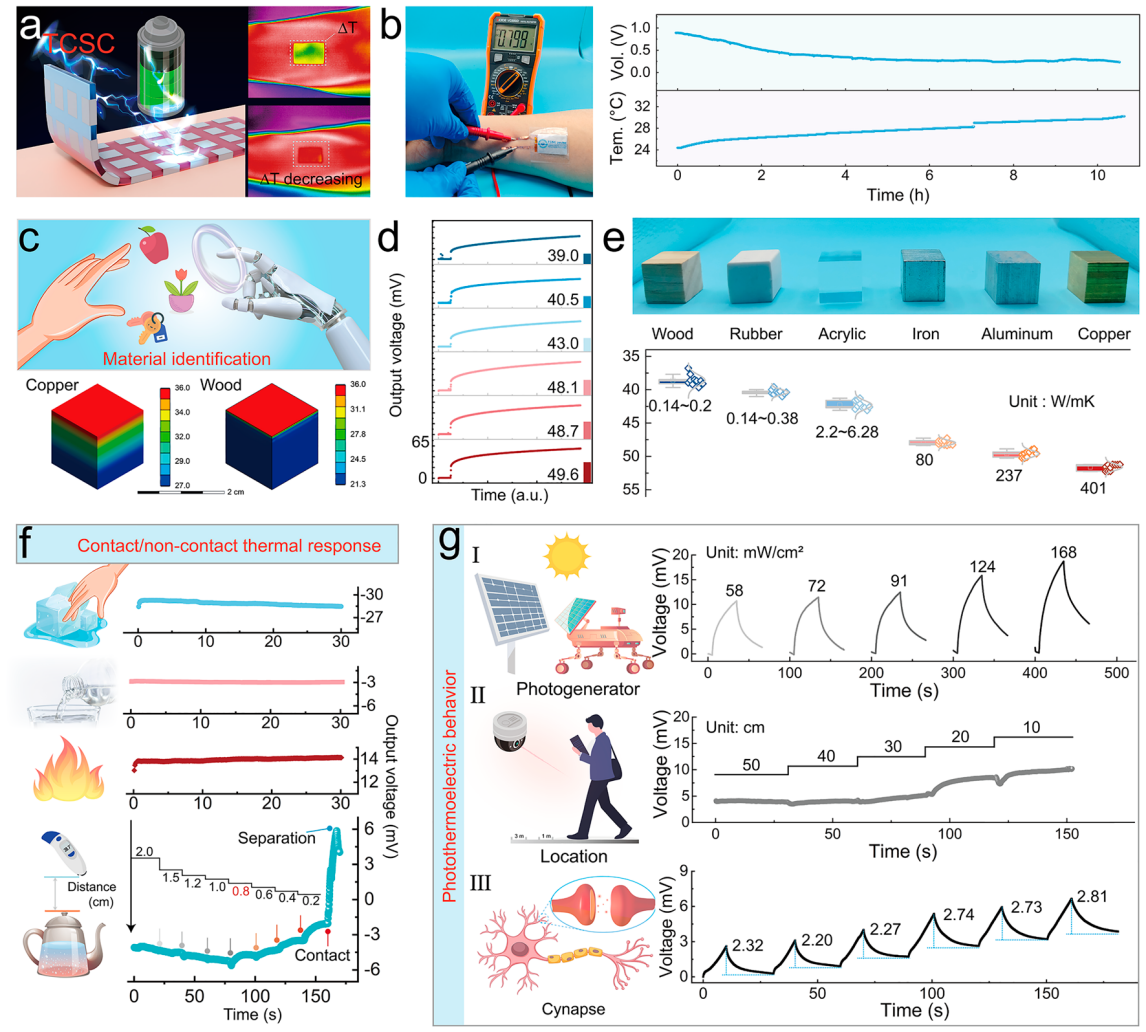
Applications of the i-TE paster in different fields. **a** i-TE paster with a multi-component series array structure is used as the thermal chargeable supercapacitor to collect the heat from the body skin. **b** After the device adheres to the skin, the output voltage of the i-TE paster is more than ten hours. **c** Schematic of material identification, and the FEA simulation on temperature field of copper and wood blocks based on the i-TE. **d** Output voltage responses of the i-TE paster to the different materials. **e** Data statistics under multiple identification and summary of thermal conductivity of different materials. **f** Contact/non-contact temperature detection function of the i-TE paster can be used to prevent objects with high/cold temperatures from harming people. **g** Application of the i-TE paster based on photo thermoelectric behavior

Ultimately, some application fields are further developed to meet different usage scenarios based on the designed i-TE paster. Firstly, the i-TE paster can be used as TCSC to collect the heat from the body's skin, convert it to electricity, and finally store energy, which is based on the constant temperature difference between the human skin and the external environment. Involving the storage performance of the device, a detailed description and data are shown in Note S6. The i-TE paster with a multi-component series array structure was fabricated and shown in Fig. S28, and each device contains six i-TE cells, which takes the additive effect of the thermal voltage into consideration. By the infrared thermal imaging instrument, as shown in Fig. 5a, a distinct area of temperature difference can be seen when the paster was just applied to the arm, and based on the thermal conduction phenomenon, there is a gradual decrease in temperature difference. Subsequently, the largest thermal voltage was output when the beginning of the paster came into contact with the skin (Fig. 5b). Then it can be found that the surface temperature of the paster monitored in real-time by a contact thermocouple rises, meanwhile, the thermal voltage output via the device decreases gradually (Fig. S29). Moreover, the self-discharging curve is adopted to study the electrochemical behavior of the paster (Fig. S30). The thermal charge of the i-TE paster at a temperature difference of 3 K, an output voltage of ~ 0.35 V can be rationally achieved. It is worth mentioning that the gradual voltage growth following the self-discharge process with a temperature of 0 K can be attributed to the chemically self-charging behaviors. A 1 KΩ resistance was loaded at both ends of the patch, there was a rapid voltage drop and then remained constant, and the related *I-t* curve is also provided.

Secondly, TE technology is capable of classifying the materials based on thermal conductivity, which promises to combine deep learning to improve the realism of VR physical sensations, as well as expand the range of haptic perception [7, 33, 34]. As shown in Fig. 5c, FEA simulations are continued to verify the difference in temperature distribution caused by different thermal conductivities based on the heat diagram. An i-TE patch prepared by a single component was attached to the fingertip, the fingertip gently touched the blocks made of a series of different materials (wood, rubber, acrylic, iron, aluminum, copper) to collect the output voltage of the patch to 20 s. Figure 5d shows the i-TE paster responses to these materials to output the different thermal voltages based on inherent heat transfer capacity supported by the different thermal conductivities. It can be found when touching the copper block, the paster generates the relative maximum voltage, while when touching the wood block, the output voltage value is smaller. Furthermore, the voltage values of ten experiments are listed in Fig. 5e, and the data statistics are carried out. Combined with the thermal conductivities of these materials, the potential application of accurate material information recognition through the i-TE paster is confirmed.

Thirdly, the skin, as the main interface for interaction with the environment, is the largest thermal sensory organ in the human body. When the skin surface touches the heated body, changes in skin temperature are detected by transient receptor potential (TRP) ion channels, which are mainly located in free nerve endings in the dermis of the skin [35]. However, once the skin comes into contact with harmful thermal stimulation, physical damage will inevitably occur. According to the thermal radiation theory, the heat energy transfer between two separated objects with different temperatures is realized through infrared radiation [36]. Thus, the temperature detection of contact and non-contact with collaborative and intelligently recognized characteristics seems meaningful and can be realized. As shown in Fig. 5f, when the fingertip with an i-TE paster touched a glass of ice water, a stable, negative voltage was output. Similarly, a smaller negative voltage was obtained by touching a glass of tap water at about 22 °C. Then, the paster generated a positive voltage after touching a glass of warm water at a comfortable temperature of about 45 °C. Therefore, the i-TE paster was proven to function as a sensitive and stable contact temperature detection and can characterize the hotness/coolness of the object being contacted based on the nature of the output voltage (positive/negative) in terms of skin temperature. Subsequently, the non-contact temperature detection behavior of the i-TE paster was further confirmed by continuously adjusting the effective distance between the heat source body and the fingertip with the device. It can be found that 0.8 cm can be considered as the maximum distance for contactless temperature perception between skin temperature and boiling water at 80 °C based on the thermal radiation effect. For the same reference voltage, the slope of the extracted voltage increases gradually with decreasing distance. Until the i-TE paster touches the heat source body, it enables it to reach the set threshold of the output voltage slope, and at the same time triggers an alarm. The process successfully simulates the intelligent temperature protection mechanism that is in the safe zone, gradually approaching the dangerous distance and arriving at the point of injury. It is worth mentioning that this rule also applies to the cases where the temperature of the heat source body continues to rise, and where a cold source that can cause touch damage is involved.

Fourthly, based on non-contact temperature detection, the photothermoelectric (PTE) technology was further developed, which can convert optical energy directly into electricity. Compared with photovoltaic (PV), PTE technology, a combination of photo-thermal effect and thermoelectric behavior, is considered to have advantages of the large energy storage capacity, low production cost, synchronous support, etc. [37, 38]. With the support of thermal energy storage, it can also achieve 24 h of continuous and stable power generation, even in the evening and rainy days. PANI, as a conventional conductive polymer, has been verified to be a remarkable photothermal material, as shown in Fig. S31. Furthermore, a transparent PI film was customized and was involved in assembling the i-TE paster. Figure 5g I displays the device response to the red light of 925 nm with different optical power, and it can be found gradient voltage outputs, which demonstrates that the paster has satisfactory PTE behavior. Moreover, under a certain power of red light, a gradient rising voltage curve was obtained by gradually shortening the distance between the device and the light source, which can be utilized to detect the distance of a target object, position, and recognize orientation (Fig. 5g II). In addition, neuro-perception and action-inspired electronics are becoming increasingly important for interactive human–machine interfaces and intelligent robots. As a result, the construction of artificial reflex arcs, such as retinal morphology eyes, functional prostheses, and tactile receptors, has become the most popular hotspot in the field of perceptual learning. PTE behavior, based on i-TE technology, has been also introduced into the field thanks to the sensitive response to light and charge storage effects. Therefore, the i-TE paster can be used as a self-powered photoreceptor synapse. As shown in Fig. 5g III, the excitation can be defined as a change in the output voltage under applied/eliminated light, and there is a series of learning-forgetting processes for each light pulse. Throughout six pulses applied, the synaptic weights showed a growth benefit, demonstrating the generalization of stimulus-aware reinforcement learning. This enhancement may be caused by a combination of the superposition of mobile ions in the system under infrared stimuli and from the charge storage effect under electrostatic forces at the electrodes, similar to the plasticity enhancement of photonic synapses fabricated from conventional heterogeneous structures.

## Conclusions

In summary, a high-performance i-TE paster based on organic gel electrolyte was developed and then applied in the fields of thermal chargeable energy storage, temperature-triggered material identification, contact/non-contact temperature detection, and photo thermoelectric conversion. Combining the polymer architecture and ionic solvation of the gel, the prepared organic gel-based electrolytes can output up to a ZT value of 0.35, which is close to that of conventional inorganic semiconductor thermoelectric materials. Relying on the PANI-C@PI electrode obtained by in situ sintering, the assembled i-TE device exhibits a stable high Seebeck coefficient of 28 mV K^−1^ and the robustness of thermal voltage output under applied stress, with a voltage output variation of less than 5% over more than 20 power generation processes, which ensures the i-TE paster can be attached to the surface of the skin to realize the detection and harvesting of heat.

## Supplementary Information

Below is the link to the electronic supplementary material.

Supplementary file1 (DOCX 4126 KB)
