# Supplementary material for: An Ultra-Thin Wearable Thermoelectric Paster Based on Structured Organic Ion Gel Electrolyte
Source: Nanomicro Lett. 2025 Mar 31;17:204. doi: 10.1007/s40820-025-01721-4 (PMC11958907; doi:10.1007/s40820-025-01721-4)
Supplement: Supplementary file 1 — Supplementary file1 (DOCX 4126 KB) [file 40820_2025_1721_MOESM1_ESM.docx]

Supporting Information for

**An Ultra-Thin Wearable Thermoelectric Paster Based on Structured Organic Ion Gel Electrolyte**

Zhijian Du^1^, La Li^1^* and Guozhen Shen^1^*

^1^ School of Integrated Circuits and Electronics, Beijing Institute of Technology, Beijing 100081, P. R. China

*Corresponding authors. E-mail: [lali@bit.edu.cn](mailto:lali@bit.edu.cn) (La Li), [gzshen@bit.edu.cn](mailto:gzshen@bit.edu.cn) (Guozhen Shen)

**Note S1 Experimental design of histological analysis of epidermal layer of mouse skin**

Purpose of experiment: To verify whether the i-TE paster can cause inflammation of the skin epidermis when it sticks to the skin.

Subjects: A four-month-old New Zealand rabbit.

Experimental equipment and reagents: i-TE paster; H&E dye.

Experimental procedure: Firstly, the i-TE pasters were sticks on the skin of the back and abdomen of mouse respectively. Secondly, the mouse was allowed to wear for 48 h while living freely. Finally, the skin tissues of the backs and abdomens of the mouse, that were exposed to and were not exposed to i-TE pasters, were sampled to make pathological sections. After H&E staining, the pathological sections were observed under a microscope. It is worth mentioning that H&E images of skin tissues not exposed to i-TE paster served as the reference group.

**Note** **S2** **FEA simulations for the polymer electrolyte membrane**

Figure 1g demonstrates the advantages of choosing the “onion epidermal cells” structure from the perspective of heat transfer and thermal strain via FEA simulations, respectively. Firstly, some simulations of heat transfer behavior are shown in **Fig. Note**1 when applying a fixed 36 ℃ to the upper end, and applying three different temperatures (34, 33, and 31 ℃) to the lower end of the polymer membrane in sequence. It can be found a distinct gradient temperature field is presented in the three scenarios. Heat from the upper end of the applied higher temperature is gradually transferred to the bottom, producing a temperature difference at equilibrium, according to Formula S1, the magnitude of this temperature difference is affected by the thermal conductivity κ of the materials.

$\text{κ∆T=-}\frac{\text{Q}}{\text{A}}$ (S1)

where $\text{∆T}$ is the temperature difference between the upper and the bottom of the polymer membrane (K), Q is heat (J), A is the effective area (m^2^), and $\text{κ}$ is the thermal conductivity of materials (W/m⸱K).

For the designed organic gel, at the micro level, the factors that determine the thermal conductivity behavior can be attributed to the thermal excitation vibration of the polymer skeleton and the diffusion motion of heat in solution under the action of molecular collision [S1-S4]. Since solids are naturally more efficient at heat transfer than liquids, FEA simulations also confirmed that the heat conduction behavior occurring in the electrolyte gel is mainly through the pore wall and the intersection region connecting the individual pores.

In addition, the use of a polymer-supported "onion epidermal cell" structure as the skeleton of the electrolyte membrane for assembling the ionic thermoelectric device has proven to have attractive advantages. For instance, the polymer membrane with a large number of pores can absorb more electrolytes for ion diffusion movement, so that a higher density of ions can accumulate at the cold end. Moreover, the solvent in pores exhibits molecular expansion behavior when applying a high temperature, which leads to the entropy increase at the hot end of the electrolyte, and the strong disorder realizes the more active movement of ions to the cold end [S5, S6]. And, as shown in **Fig. Note**2, PEO-doped PVDF-HFP membrane was also confirmed to induce pore expansion at high temperatures, which is attributed to PEO, as a kind of thermal phase change polymer, which undergoes thermal relaxation of molecular chains at high temperatures [S7, S8]. The increase in pore volume promotes the high activity of ions in pores at the hot end of the membrane, thus achieving an output of satisfactory thermoelectric performance.


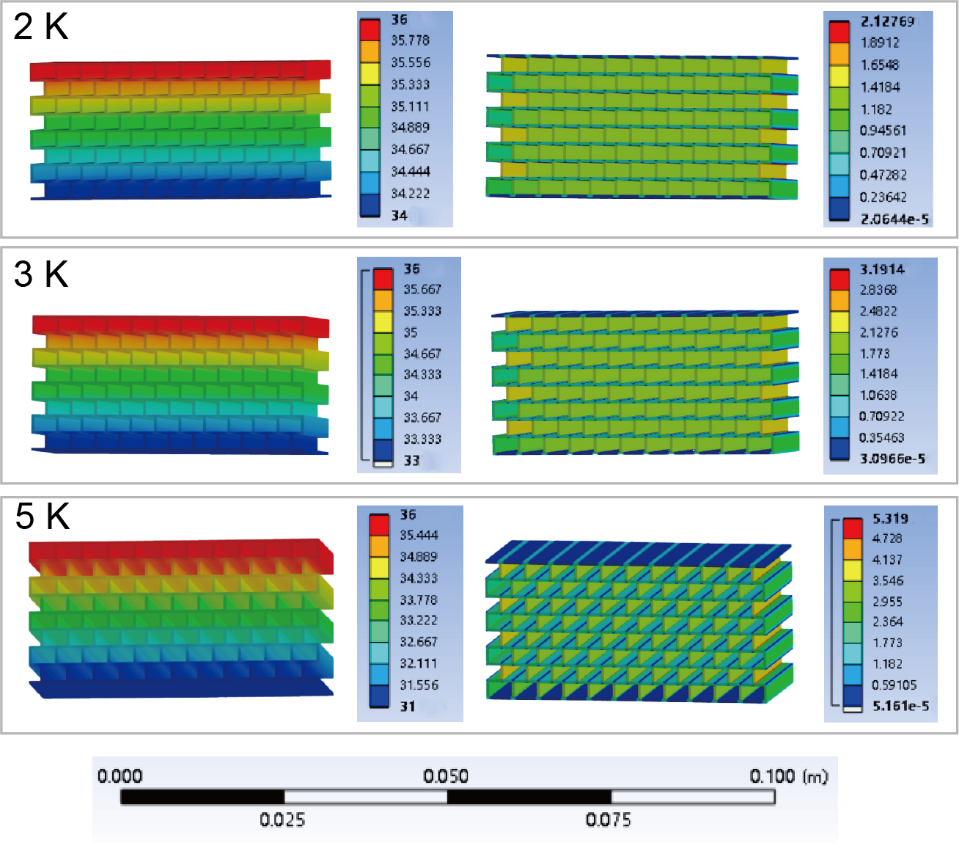


**Fig. Note1** FEA simulations of heat transfer behavior in the polymer membrane under the action of 2, 3, and 5 K temperature differences applied at both ends


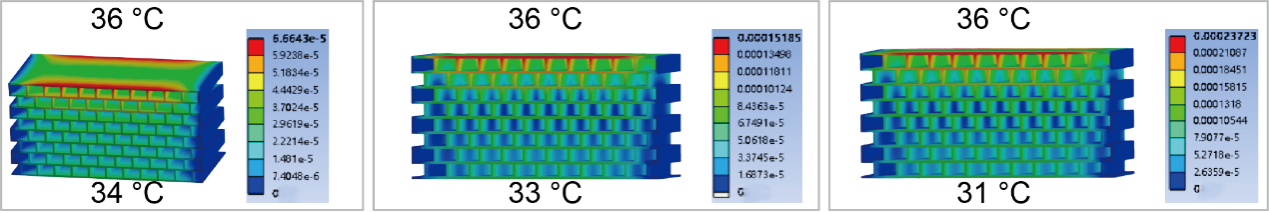


**Fig. Note2** FEA simulations of thermal stress phenomenon in the polymer membrane under the action of 2, 3, and 5 K temperature difference applied at both ends

**Note S3 Construction and principle of a homemade cold and heat supply platform**

The design of a simple homemade vertical cold/heat supply platform is shown in the **Fig. Note**3, which consists of a hot sheet driven by a DC power supply as a heat source, a Peltier cooling element as a cold source, and both the heat and cold sources are implanted with a temperature sensor to detect the temperature difference between the contact device ends. To keep the output low temperature of the cold source at a stable state, an integrated cooling system of water cooling and air cooling is provided at the bottom of the cold plate to maintain the timely dissipation of the heat generated by it. To maintain the stability of the temperature supplied to the device, the device is encapsulated in the hollow groove of the poured PDMS. The reason why PDMS is used as a fixed layer for heat isolation is because of the low thermal conductivity of PDMS, and more importantly, it has elasticity, which can easily cooperate with the slide system at the top of the heat source to achieve a tight and non-extrusion adhesion of the hot sheet to the device.


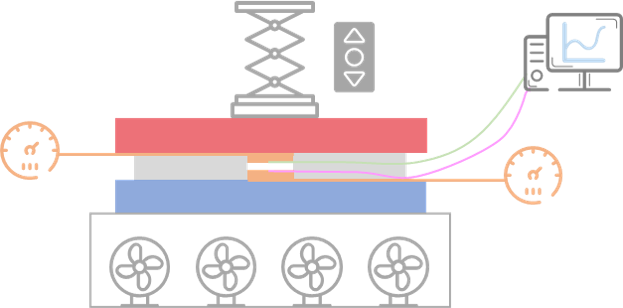


**Fig. Note3** Schematic diagram of the homemade vertical cold/heat supply platform

**Note S4** **Dielectric constants and loss Angle tangents of PVDF-HFP and its copolymer membrane**

Dielectric constant and dielectric loss angle tangent are two important physical parameters that describe the behavior of materials in the electric field. Dielectric constant ε is a macroscopic physical quantity that characterizes the ability of a dielectric to polarize and store electrical charges. It represents the amount of electrostatic energy stored per unit volume in a unit electric field [S9]. As shown in **Fig. Note**4, PVDF-HFP-PEO copolymer organized exhibits a more outstanding ε, which can be attributed to the absorption of more electrolyte or PEO doping with abundant hydrogen bonds. This high ε represents a strong ability to bind the internal charge, as well as good stability in the electric field [S10]. Moreover, it also suggests the strong bonding ability between the electrolyte and the electrode, which realizes that ions in the aggregated state are easy to induce more abundant opposite charges on the electrode surface. And, occurred in the electrolyte, a more pronounced ionic solvation phenomenon can be found due to the strong polarity, which enables more free ions in the system to undergo directed migration and thermal diffusion under the action of electric fields.

**
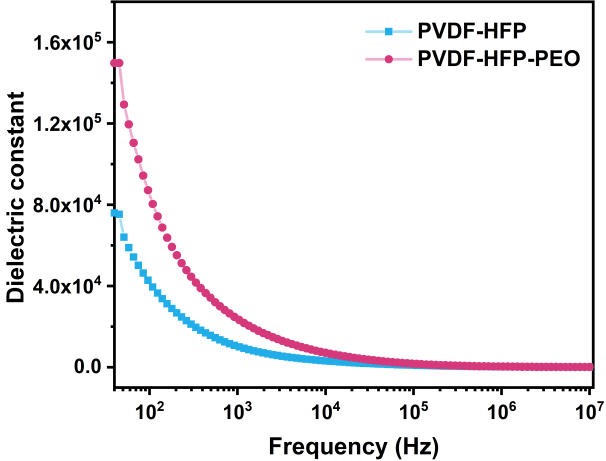
**

**Fig. Note4** Dielectric constants of PVDF-HFP and PVDF-HFP-PEO membranes

**Note** **S5 Molecular dynamics calculation**

**Computational methods:**

Quantum chemical calculations were carried out using the density functional theory (DFT) method of the Dmol3 module with B3LYP functional, custom Grimme DFT-D parameters, and DNP 4.4 basis set [S11, S12]. The k-points were set as Gamma (1 × 1 × 1) and the convergence tolerance was set as 1.0 × 10^-5^ Ha, 2.0 × 10^-3^ Ha Å ^-1^, and 5.0 × 10^-3^ Å for energy, maximum force, and maximum displacement, respectively.

The Forcite module in Material Studio software was employed to conduct Molecular Dynamic (MD) simulations at 298 K for different electrolytes [S13]. The 1M LiTf DMC: EC=2:1 electrolyte model consisted of 190 DMC, 86 EC and 20 LiCF_3_SO_3_ molecules (**Fig. Note**5a); The 1M LiTf DMC: EC=1:2 electrolyte model consisted of 92 DMC, 188 EC and 20 LiCF_3_SO_3_ molecules (**Fig. Note**5b); The 2M LiTf DMC: EC=1:2 electrolyte model consisted of 92 DMC, 188 EC and 40 LiCF_3_SO_3_ molecules (**Fig. Note**5c). The COMPASS force field was utilized for all MD simulations with a fixed time step of 1 fs (femtosecond). The systems experienced at least 5 ps (picosecond) equilibration steps in the NPT ensemble using the Berendsen barostat to maintain 0.1 GPa pressure with 0.1 ps decay constant. After the equilibration steps, the production runs were carried out in an NVT ensemble of 200 ps. All the steps used a Nosé thermostat with target temperatures of 298 K [S14]. The simulation time was long enough to ensure the electrolyte system was in equilibrium.

**
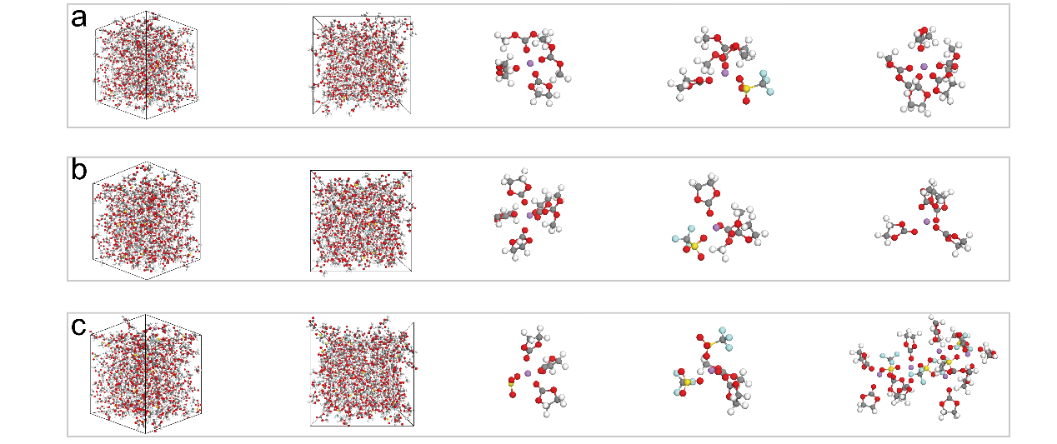
**

**Fig. Note5** Molecular Dynamic (MD) simulations of the three electrolyte models (**a**: 1M LiTf DMC: EC=2:1; **b**: 1M LiTf DMC: EC=1:2; **c**: 2M LiTf DMC: EC=1:2)

**Discussion**

As shown in Figs. 3c~g, according to molecular dynamics, a series of simulations and calculations were carried out to illustrate the ionic solvation in an ionic organic gel-based electrolyte. At the beginning, the molecular electrostatic potentials were simulated to determine the charge distribution on the molecular surface, and then affect the strength and direction of the intermolecular force. The three systems were focused and their electrostatic potential (ESP) were shown in Fig. 3c (Ⅰ represents1 M LiTf DMC: EC=1:2 system; Ⅱ represents 2 M LiTf DMC: EC=1:2 system, Ⅲ represents 1 M LiTf DMC: EC=2:1 system). As shown in Fig. 3c Ⅰ, due to the electrostatic interaction between the O atom and the Li^+^ ion, the nucleophilic sites on the DMC and EC molecules are close to the central atom. The strong electron-absorbing group -CF_3_ exists in LiTf salt of anion CF_3_SO_4_^-^, which attracts electrons on DMC and EC molecules. And in Fig. 3c Ⅱ, as the salt concentration increases, more -CF_3_ functional groups are involved in absorbing electrons from the system. In Fig. 3c Ⅲ, as the more EC molecules with more electrons involving, the electron cloud of the system becomes richer. In conclusion, in the ionic organic gel system, the solvent DMC and EC will actively attach to the Li^+^ ion due to the electronegativity and static electricity of the oxygen atom, forming a spherical shell structure (solvation), especially EC molecule with the stronger polarity. The dissociated anions in LiTf molecules contain -CF_3_, which will adsorb the electrons in the ion clusters, resulting in a decrease in the electron cloud density in the system [S15, S16].


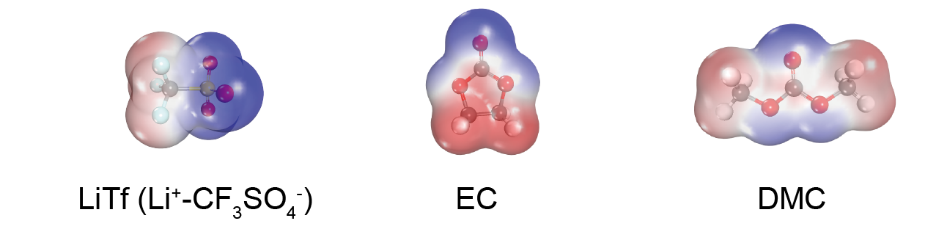


**Fig. Note6** Molecular electrostatic potentials of LiTf, EC, and DMC. As you can see from the models, nucleophilic sites are concentrated on oxygen atoms, and due to the inherent electron-pulling effect of -CF_3_ and -CH_3_, a few electrons on oxygen atoms are attracted to them

To understand the stochastic and diffuse properties of Li^+^ ion motion in electrolytes, the results of mean-squared displacement (MSD) are shown in Fig. 3d (the line with dark blue represents; the line with blue represents 2 M LiTf DMC: EC=1:2 system; the line with light blue represents 1 M LiTf DMC: EC=2:1 system). A long timescale is utilized because of the nature of diffusion motion in a time dimension, and the slopes of the obtained functions can interpret the diffusion rate by fitting the MSD curves. The linearity of the obtained MSD curves is greater than 0.99, which indicates that the ion motion has obvious randomness. Depending on the magnitude of the slopes of the line, more intense ion diffusion is thought to occur within 1 M LiTf DMC: EC=1:2 system (rate of diffusion=0.05934 Kmol/(m²·s)), which is more representative of its more prominent point conductivity. However, the 2 M LiTf DMC: EC=1:2 system is shown to have more prominent thermoelectric performance because of the large Seebeck coefficient (S=15 mV/K), which may demonstrate that the diffusion behavior of Li^+^ clusters in the system is more affected by temperature.

The Radial Distribution Function (RDF) is an important statistical tool used to describe the spatial distribution of atoms or particles in a system at a specific distance. In this system, with Li^+^ ion as the center, anions CF_3_SO_4_^-^, solvent molecules DMC and EC, based on electrostatic force and solvation, are distributed in outer space to characterize the structure of Li^+^ ion clusters. As shown in Fig. 3e~g, the blue line represents Li-CF_3_SO_4_^-^, the black line represents Li-EC, and the red line represents Li-DMC, based on the action of Li^+^-O (the corresponding coordination numbers N(r) are plotted with dashed lines). For all electrolytes, the first peak of RDF is around 2.0 Å, and the second peak appears at around 4.0 Å, which can indicate that it is easy to dissociate LiTf molecule under the action of the solvent DMC and EC. Fig. 3e reflects the structure of 1 M LiTf DMC: EC=1:2 system, and it can be found that the coordination number (CN) of Li-EC is 4.34, and Li-DMC is 1.72, indicating that each Li^+^ ion is surrounded by four EC molecules and one to two DMC molecules in the first solvation shell for tridentate. Similarly, in the 2 M LiTf DMC: EC=1:2 system (Fig. 3f), the CN value of Li-EC is 4.12, and Li-DMC is 1.82. However, the CN of Li-CF_3_SO_4_^-^ in the electrolyte dissolved 1 M LiTf is significantly lower than that in the electrolyte dissolved 2 M LiTf. The solvation structure of Li^+^ undergoes a drastic change, which results in the formation of a large number of aggregates of Li^+^ and TFSI^−^ (shown in the last picture of **Fig. Note**5). In 1 M LiTf DMC: EC=2:1 system, the CN values of Li-CF_3_SO_4_^-^, Li-EC, and Li-DMC are 3.81, 2.13, and 3.62, respectively (Fig. 3g). Compared with that in 1 M LiTf DMC: EC=1:2 system, the results demonstrate the EC, as a strong polarity, enables dissolve more LiTf salt and induce the mass generation of Li^+^ ions.

**Note S6 In-situ FT-IR of the** **gel electrolyte (1 M LiTf DMC: EC=1:2)**

We continued to demonstrate the intensity of ionic solvation depending on the temperature. First, when the temperature increases, the movement speed of solvent molecules is accelerated, and the interaction with ions is also enhanced, which helps the ions to be better solvated in the solvent [S17]. When the temperature reaches 26 ℃, the solvation intensity presents the maximum. However, as the temperature rises, the SI values gradually decline, which can be attributed to the thermal motion of solvent molecules being accelerated, the interaction force between them is weakened, and the interaction force between solvent molecules and ions is also affected. As the solvent molecules move more vigorously, their alignment around the ions may become less tight, resulting in a reduction in solvation strength [S18]. Therefore, when the i-TE device works under applying temperature differences, the low ionic solvation at the hot end induces more Li^+^ to diffuse to the cold end in the state of free ions or multiionic complex, and then the electrostatic coordination effect occurs with the active site on the electrode surface, resulting in a decrease in the surface potential of the electrode, thus achieving the generation and output of the electromotive force.

**
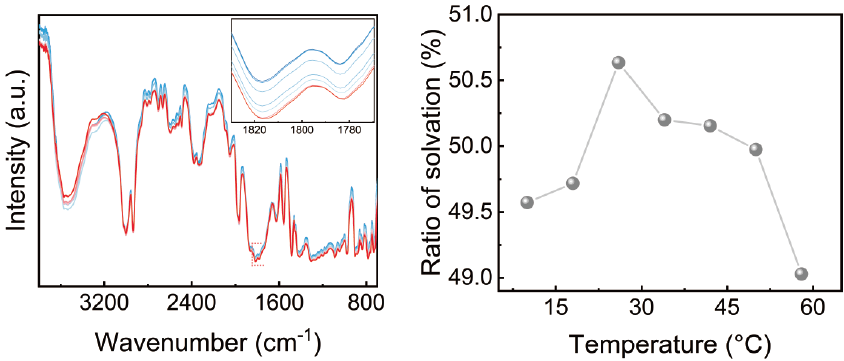
**

**Fig. Note7** In situ FT-IR of the gel electrolyte (1 M LiTf DMC: EC=1:2)

**Note S7 Characterization of capacitive properties of i-TE paster as TCSC and calculation of thermoelectric conversion efficiency**

In practice, the i-TE device can be used as the TCSC because of the sandwich structure and the electric charge generated on the electrode surface based on the electric double layer (EDL) effect. Therefore, on the one hand, this note aims to characterize the basic electrochemical properties of the device, such as the capacitance (C); On the other hand, the energy density (E), power density (P), and thermoelectric conversion (η) efficiency were calculated to further evaluate the electrical properties of the prepared thermal-rechargeable supercapacitor (TCSC). According to the cyclic voltammetry (CV) curves shown in Figs. **Note**8a, b, it can be found that the TCSC assembled with PANI-C@PI electrode film has the most outstanding capacitivity and conductivity, compared with the other three films. A considerable rate capability is shown in Figs. **Note**8c, d. As shown in Figs. **Note**8e, f, as the temperature difference applied to the upper and lower electrodes of the TCSC increases gradually, the capacitance of the device also increases simultaneously, and the temperature difference to area ratio capacitance curve has a linear relationship (slope=0.55592 µF/cm^2^⸱K), which proves the thermal charging behavior of the TCSC and the satisfactory temperature difference following phenomenon.


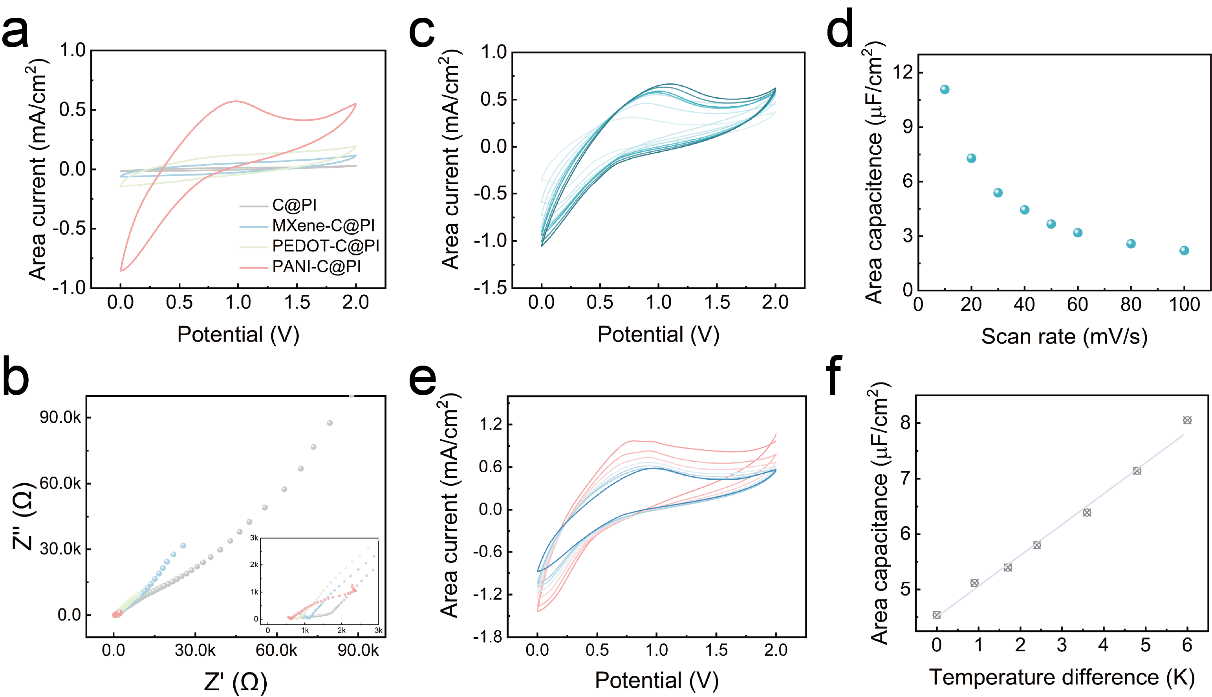


**Fig. Note8 The** **electrochemistry performances of TCSC device.** (**a~b**) The cyclic voltammetry (CV) curves (**a**) and Nyquist plots (**b**) of the TCSCs assembled with C@PI, MXene-C@PI, PEDOT-C@PI, PANI-C@PI electrode films. (**c~d**) CV curves (**c**) and the area capacitance calculated (**d**) of the TCSC assembled with PANI-C@PI electrode film at different scan rates. (**e~f**) CV curves (**e**) and the area capacitance calculated (**f**) of the TCSC assembled with PANI-C@PI electrode film at different temperature differences

Through testing and calculation, the conductivity of TCSC is 12.5 mS/cm, the capacitance is 5.6 µF, the Seebeck coefficient is 28 mV/K, the cross-sectional area of the electrolyte is 0.189 mm^2^ and the thermal conductivity coefficient is 0.04 W/m⸱K, relatively. The energy conversion efficiency (η) can be obtained from E/Q, where E and Q are the electric energy stored in the device (**Fig. Note**9a) and the quantity of heat provided (**Fig. Note**9b) to the device. According to the formula $\text{E=Cd}\text{V}^{\text{2}}\text{/2}$, where C is the capacitance, and V is the output thermal voltage of the TCSC. Q was obtained from the formula $\text{Q=}\text{κ}\text{AdtdT}\text{/l}$, where κ, A, l, dt, and dT are the effective thermal conductivity, the cross-sectional area of electrolyte, length of the electrode, thermal-charging time, the temperature difference between the hot and cold side of the electrolyte, respectively. Therefore, as shown in **Fig. Note**9c, η_0.5 K_=0.2%, η_1.1 K_=0.25% and η_6 K_=1.3%. Moreover, in **Fig. Note**9d, the power density P of TCSC can be obtained according to the formula $\text{P=E/Adt}$, where P_0.5 K_=0.0057 W/m^2^, P_1.1 K_=0.015 W/m^2^ and P_6 K_ =0.45 W/m^2^.


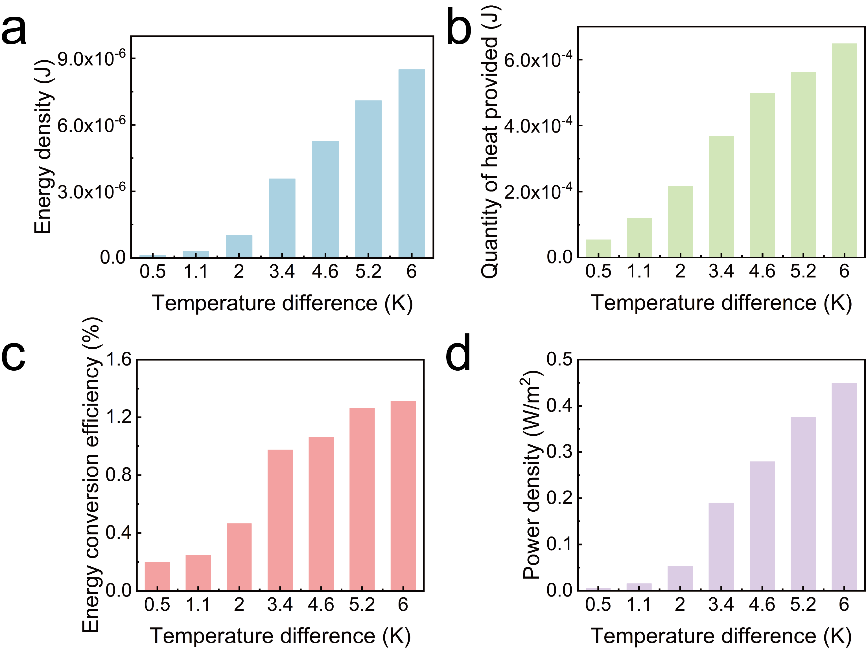


**Fig. Note9** The energy density (**a**), the quantity of heat provided (**b**), energy conversion efficiency (**c**), and power density (**d**) of the TCSC, under applying the different temperature differences

**Note S8 Experimental design of photo thermoelectric behavior of i-TE device**

**
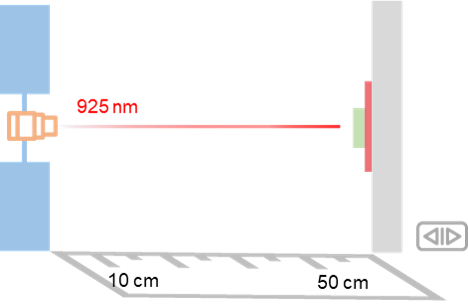
**

**Fig. Note10** Schematic diagram of the homemade infrared test platform

A simple homemade infrared test platform was designed to demonstrate the ability of i-TE device to respond to infrared light and is shown in **Fig. Note**10. The platform consists of a 925 nm laser light source with adjustable light intensity, and the device (green) is fixed on a hot sheet (supplied at 37 °C) for the simulator to be attached to the human skin surface. In this study, we studied the output voltage of the device under different light intensity. And the device end is moved by the slide rail to demonstrate the function of measuring distance of the objects.

**Note S9 Experimental** **procedure for determining thermal conductivity of** **membrane materials by laser flash method**


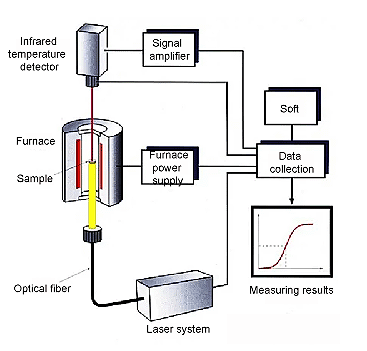


**Fig. Note11** Schematic diagram of the thermal conductivities of materials determined by laser flash method

A universal method to determine the thermal conductivities of membrane materials was employed, which was further used to calculate the ZT figures.

Test Instrument: Laser thermal conductivity meter (NETZSCH LFA 457)

Testing Principle: The surface of the sample is heated by laser pulses and the temperature change at the back of the sample is measured using an infrared detector to calculate the thermal diffusion coefficient (α), which is then combined with the specific heat capacity (C_p_) and density (ρ) to derive the thermal conductivity (λ).

Sample Preparation: The sample should be flat, uniform, and of moderate thickness (typically a few tens of microns to a few millimeters). The surface should be clean and, if necessary, coated with a graphite layer to enhance laser absorption.

Sample mounting: Place the sample in the sample holder and ensure that the laser beam is directed perpendicularly to the sample surface.

Parameter setting: Set the laser energy, pulse width, sampling frequency and other parameters.

Test Run: Start the test, the laser pulse heats up the sample, the infrared detector records the temperature change on the backside.

Data Acquisition: Record the data of temperature change over time.

Data analysis: Use the software to analyze the data, calculate the thermal diffusion coefficient, specific heat capacity and density, and finally derive the thermal conductivity.

Using an infrared detector to continuously measure the corresponding temperature rise process in the center part of the upper surface of the sample, the temperature rise-time relationship curve is obtained, by measuring the half temperature rise time t_1/2_ (defined as the time required for the temperature of the upper surface of the sample (detector signal) to rise to half of the maximum value after receiving the laser pulse irradiation), by the formula: α = 0.1388 × d^2^ / t_1/2_ (d: the thickness of the sample) The thermal diffusion coefficient α of the sample at temperature T can be obtained. And then by the thermal conductivity and thermal diffusion coefficient of the conversion relationship: λ_(T)_ = α_(T)_ × Cp_(T)_ × ρ_(T)_, at a known temperature T under the thermal diffusion coefficient α, the specific heat Cp and the density of ρ can be calculated under the circumstances of the thermal conductivity. And conversely, the thermal conductivity λ of the material can be calculated by specific heat Cp and density ρ. The density ρ was obtained by a true density tester. And the specific heat Cp was obtained by reference samples:

The specific heat Cp is defined by the formula: Cp = Q / m × ∆T (Cp unit: J/(g⸱K); Q, absorbed heat, unit J; ∆T, the sample absorbed heat temperature rise, unit K). When the test in the light energy is the same, samples and specimens of the lower surface of the absorption area and absorption ratio are the same: Q_specimen_ = Q_sample_.

**Supplementary Figures**


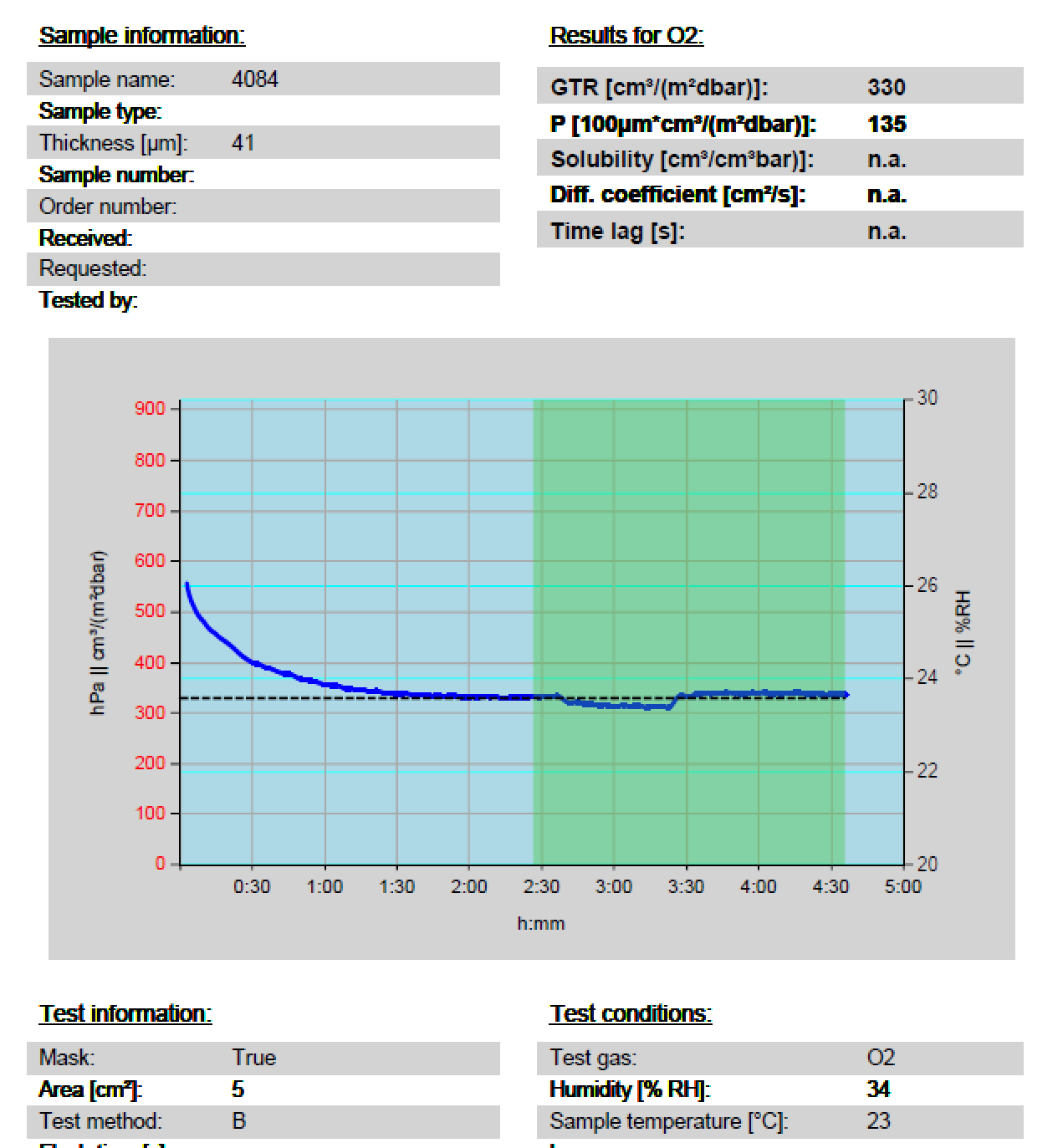


**Fig. S1** The oxygen permeability of the encapsulation material in the i-TE paster

The gas permeability coefficient refers to the volume of gas passing through the sample per unit area and per unit time under constant temperature/humidity (23.1 ℃, 51%) and pressure difference. It is usually expressed as a volume value at standard temperature and 1 atmosphere, in units of cm^3^/(m^2^⸱24h⸱0.1MPa). The encapsulated membrane was cut into a specific size and tightly fixed in the contact groove of the low-pressure chamber and the high-pressure chamber. The high-pressure chamber was filled with about 0.1 MPa of oxygen. The pressure measuring unit tests the pressure increment in the closed system and calculates the functional gas amount in units of time. The experimental results show that the oxygen permeability of the membrane reaches 330 cm^3^/(m^2^⸱24h⸱0.1MPa), which demonstrates that i-TE paster will not affect the normal breathing of the skin after sticking on human skin, and has considerable wearing comfort.


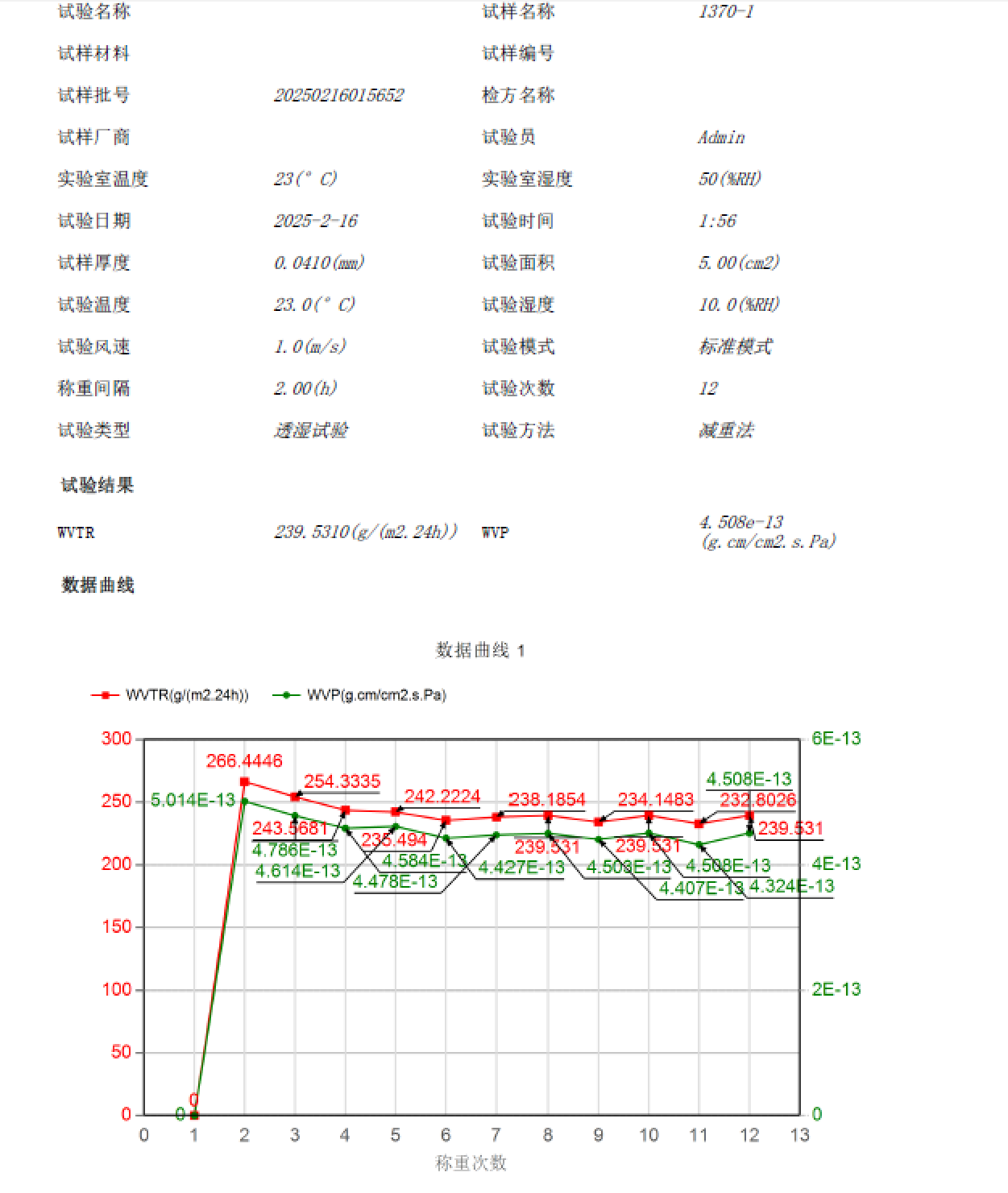


**Fig. S2** The water permeability of the encapsulation material in the i-TE paster

Water vapor transmission rate (WVTR) is the amount of water vapor passing through a material per unit area per unit time and is an important indicator to evaluate the comfort of skin wearable devices. WVTR of the encapsulated membrane in i-TE paster was measured by the weight loss method in a room temperature environment (temperature: 23.1 °C, relative humidity: 51%), in which the test wind speed was 1 m/s and the weighing interval was 2 h. Finally, the stable and outstanding WVTR of 239.531 g/(m²⸱24h) represents that the encapsulated membrane presents polyporous, which contributes to the water vapor permeation.

**
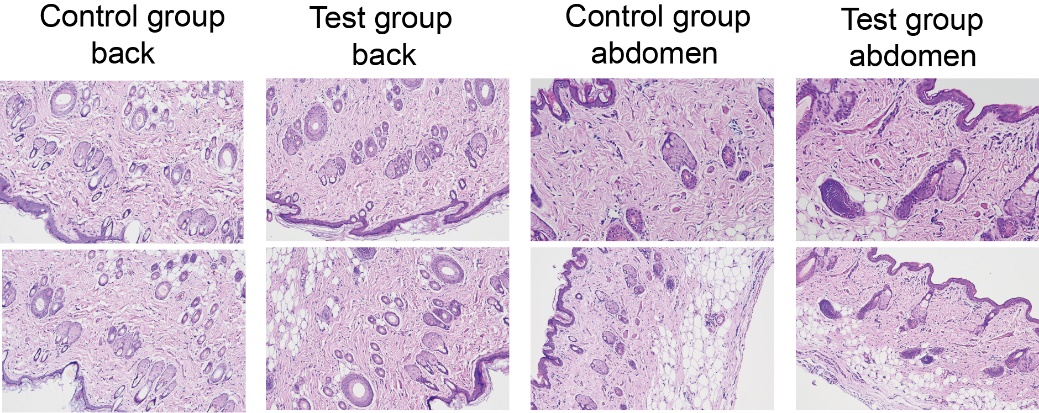
**

**Fig. S3** H&E images of the back and abdomen after wearing the i-TE pasters for 48 hours

***
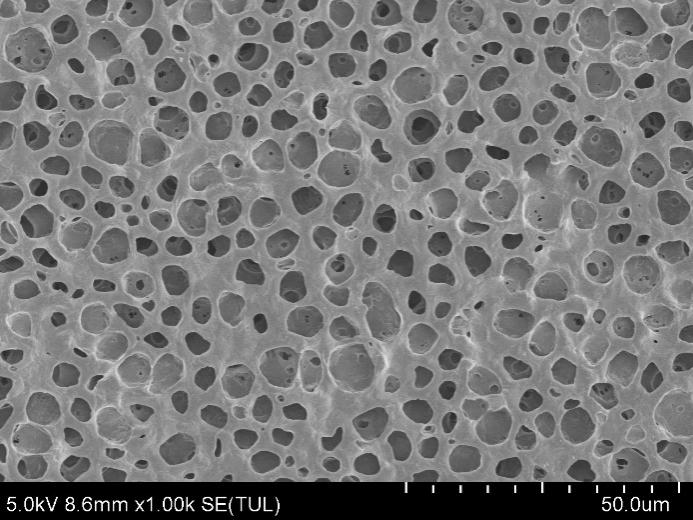
***

**Fig. S4** SEM image of the surface of PVDF-HFP-PEO membrane

*
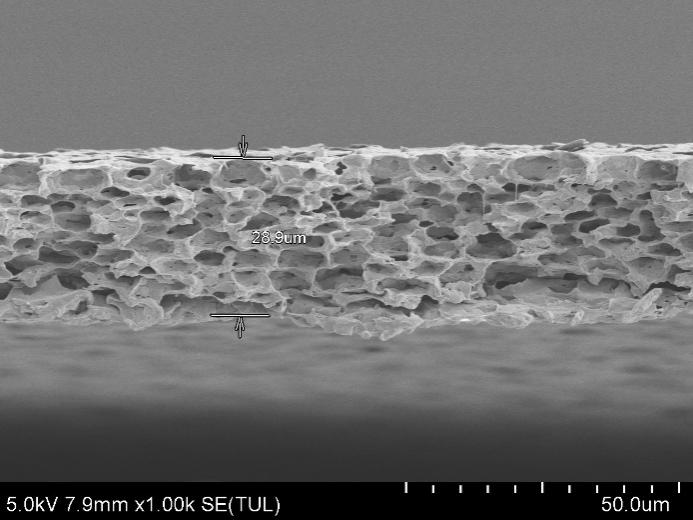
*

**Fig. S5** SEM image of the section of PVDF-HFP membrane

**
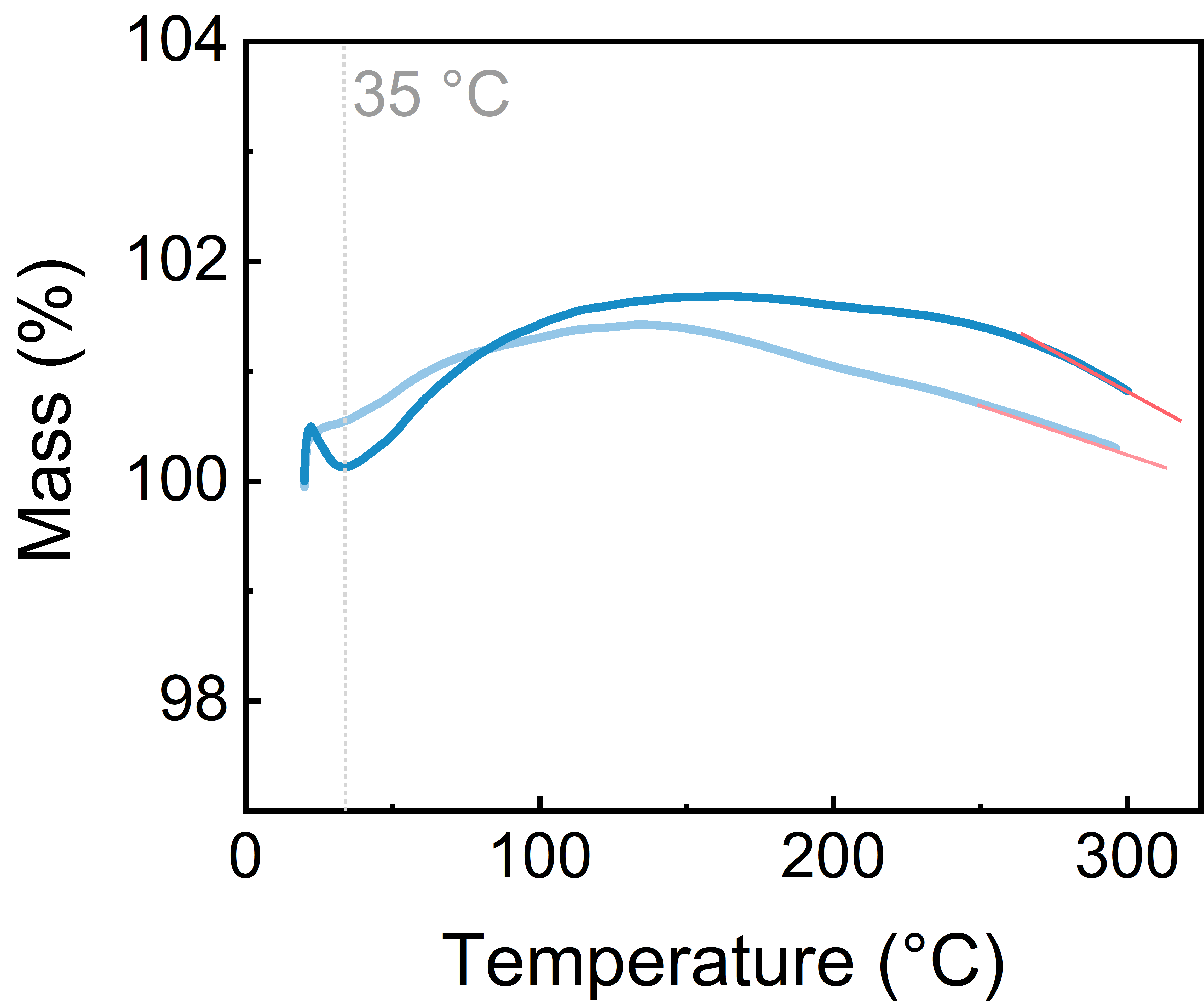
**

**Fig. S6** TG curves of PVDF-HFP (light) and PVDF-HFP-PEO (gel) membranes

Thermogravimetric (TG) testing can indirectly show the thermal expansion behavior of porous polymer films. For example, as shown in Fig. S6, PVDF-HPF-PEO membrane presents a rapid loss of mass when they are just heated, due to significant thermal expansion of the pores within the membrane, which results in the release of internal gases. During heating, both PVDF-HFP and PVDF-HFP-PEO membranes show apparent weight gain, i.e., there is no mass change in the sample itself, but the change in gas buoyancy caused by the change in temperature causes the sample to show an increase in mass with temperature. This apparent weight gain is strongly due to the very light membrane material, and the more pronounced apparent weight gain exhibited by the composite membrane can be attributed to the more outstanding mass loss as the internal gas is released. In addition, the expanded pores lead to a deterioration in the overall thermal stability of the porous polymer membrane as the internal structure becomes loose or cracked, which may promote the decomposition of the membrane at elevated temperatures. Therefore, PVDF-HFP-PEO membrane exhibits an increased rate of mass loss above 250 °C.


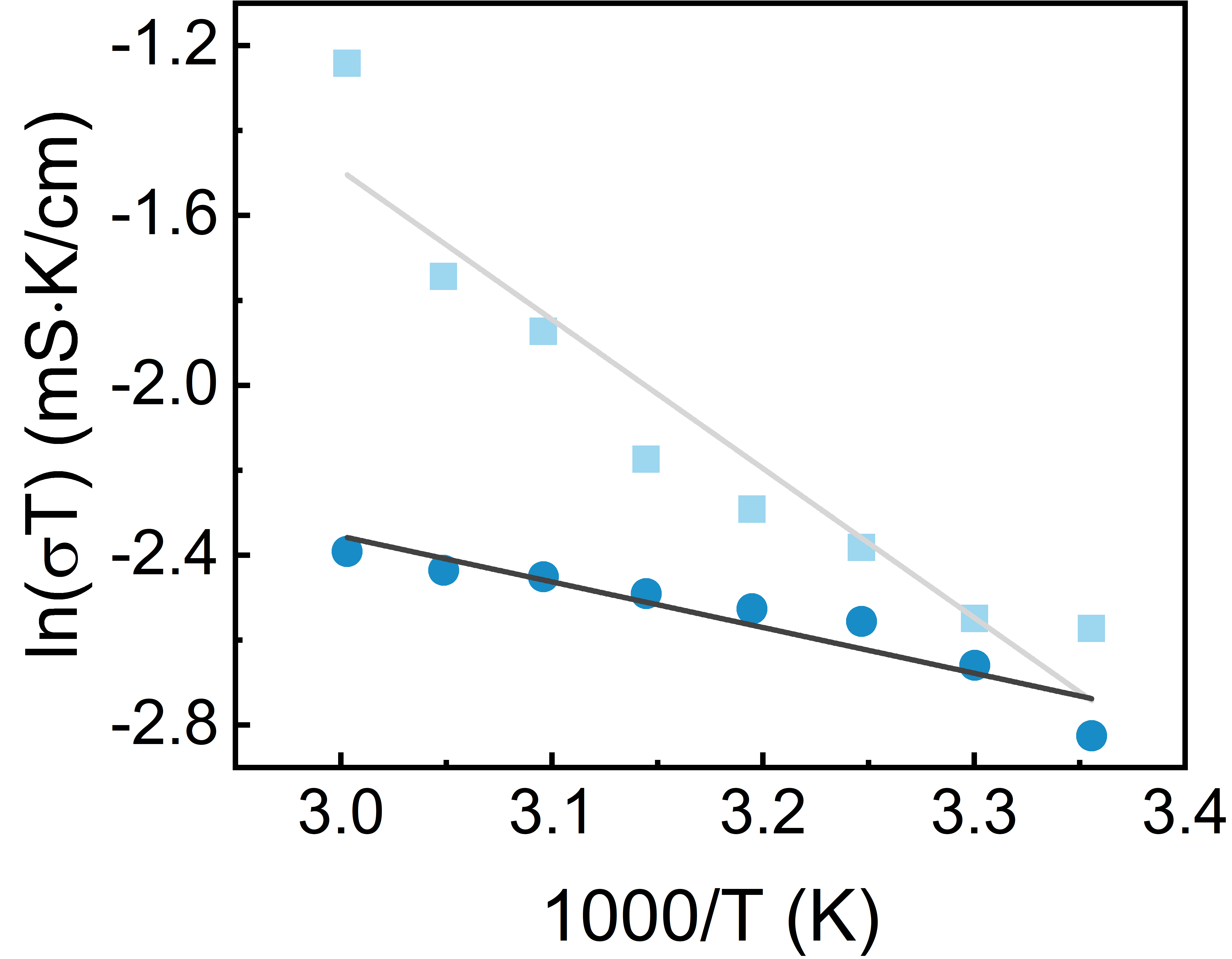


**Fig. S7** The temperature-dependent Arrhenius fit curves of PVDF-HFP and PVDF-HFP-PEO gels

A series of resistances with temperature-dependent were obtained by EIS and R_ct_s were further obtained after intraducing the equivalent circuit. The their ionic conductivities were alculated by Eq. $\text{σ=}\frac{\text{l}}{\text{A⸱}\text{R}_{\text{ct}}}$ (l : thickness/cm; A: effective area/cm2; R_ct_: ion migration impedance/mS). The Arrhenius relation was employed to fit the ion migration potential (E_a_), which represents as slope. The slope presents larger, Ea value is prominent, and the ion migration is limited. As shown in Fig. S7, the PVDF-HFP-PEO gel exhibited more active ion migration.

*
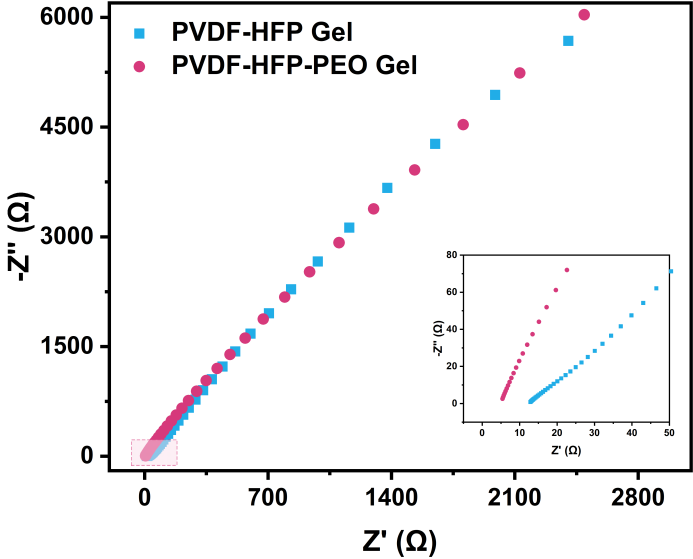
*

**Fig. S8** Nyquist plots of PVDF-HFP and PVDF-HFP-PEO gels


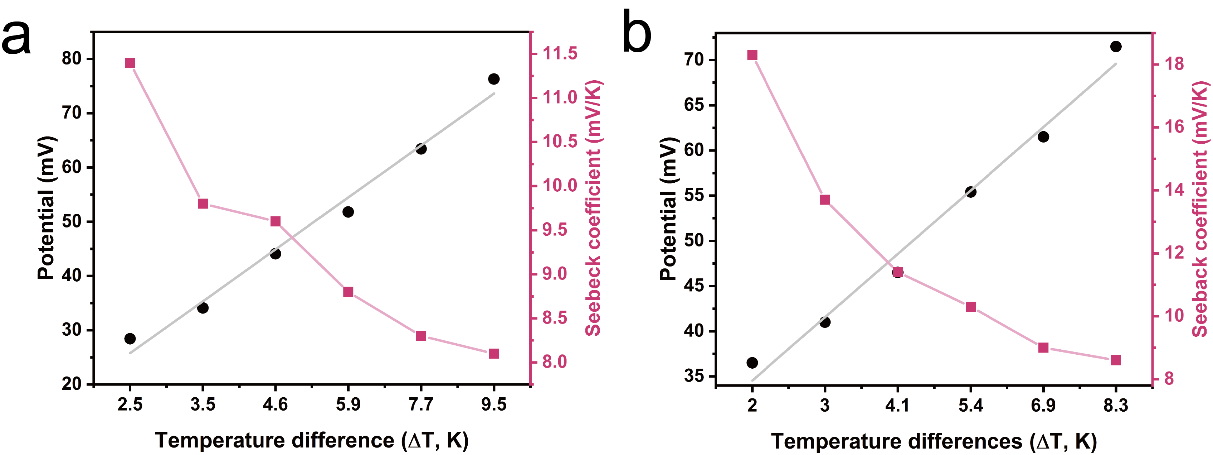


**Fig. S9** The potentials and Seebeck coefficients of PVDF-HFP (**a**) and PVDF-HFP-PEO (**b**) gels under different temperature differences


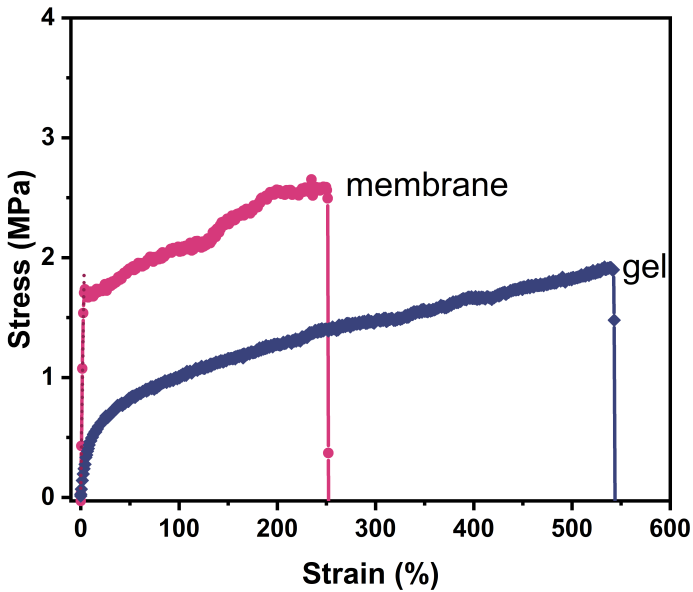


**Fig. S10** The stress-strain curves of PVDF-HFP-PEO membrane and gel

*
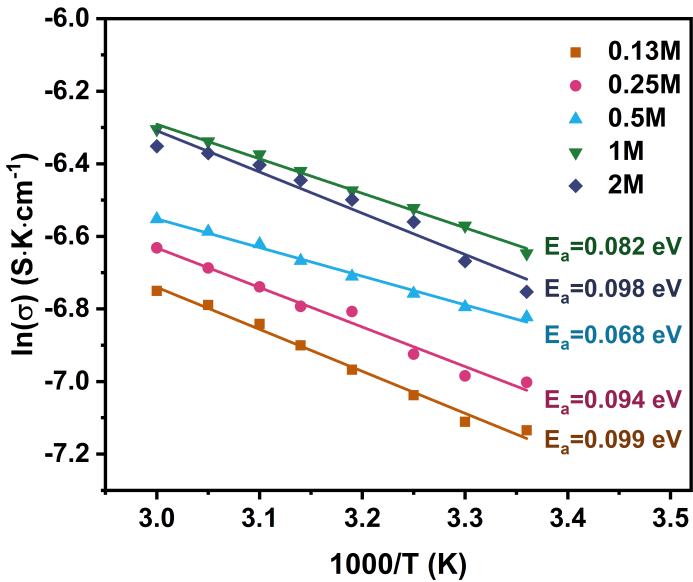
*

**Fig. S11** The temperature-dependent Arrhenius fit curves of PVDF-HFP-PEO gels with different content of LiTf salt


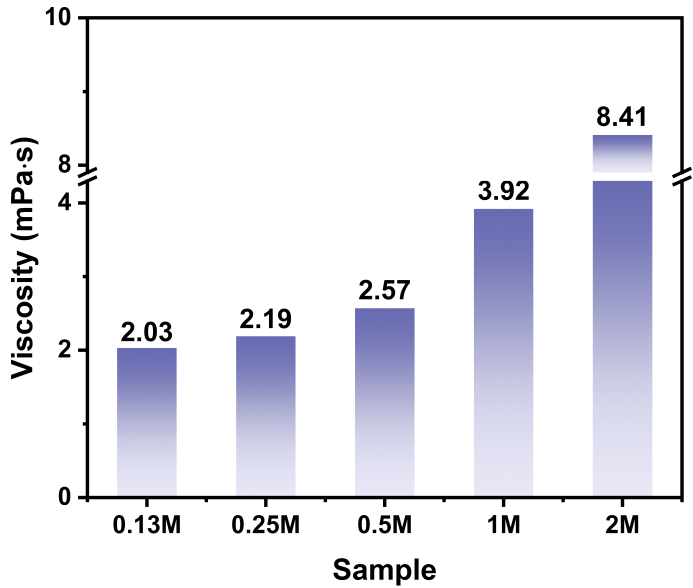


**Fig. S12** Viscosities of PVDF-HFP-PEO gels with different content of LiTf salt


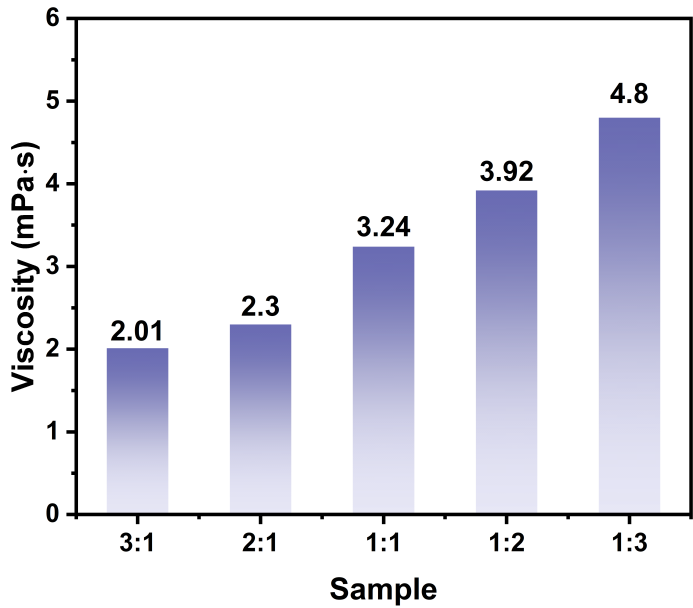


**Fig. S13** Viscosities of PVDF-HFP-PEO gels with different ratios of DMC:EC

*
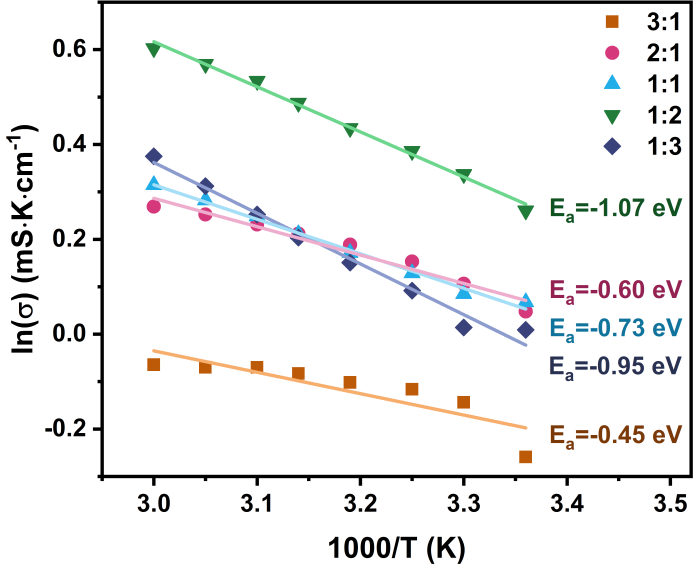
*

**Fig. S14** The temperature-dependent Arrhenius fit curves of PVDF-HFP-PEO gels with different ratios of DMC:EC


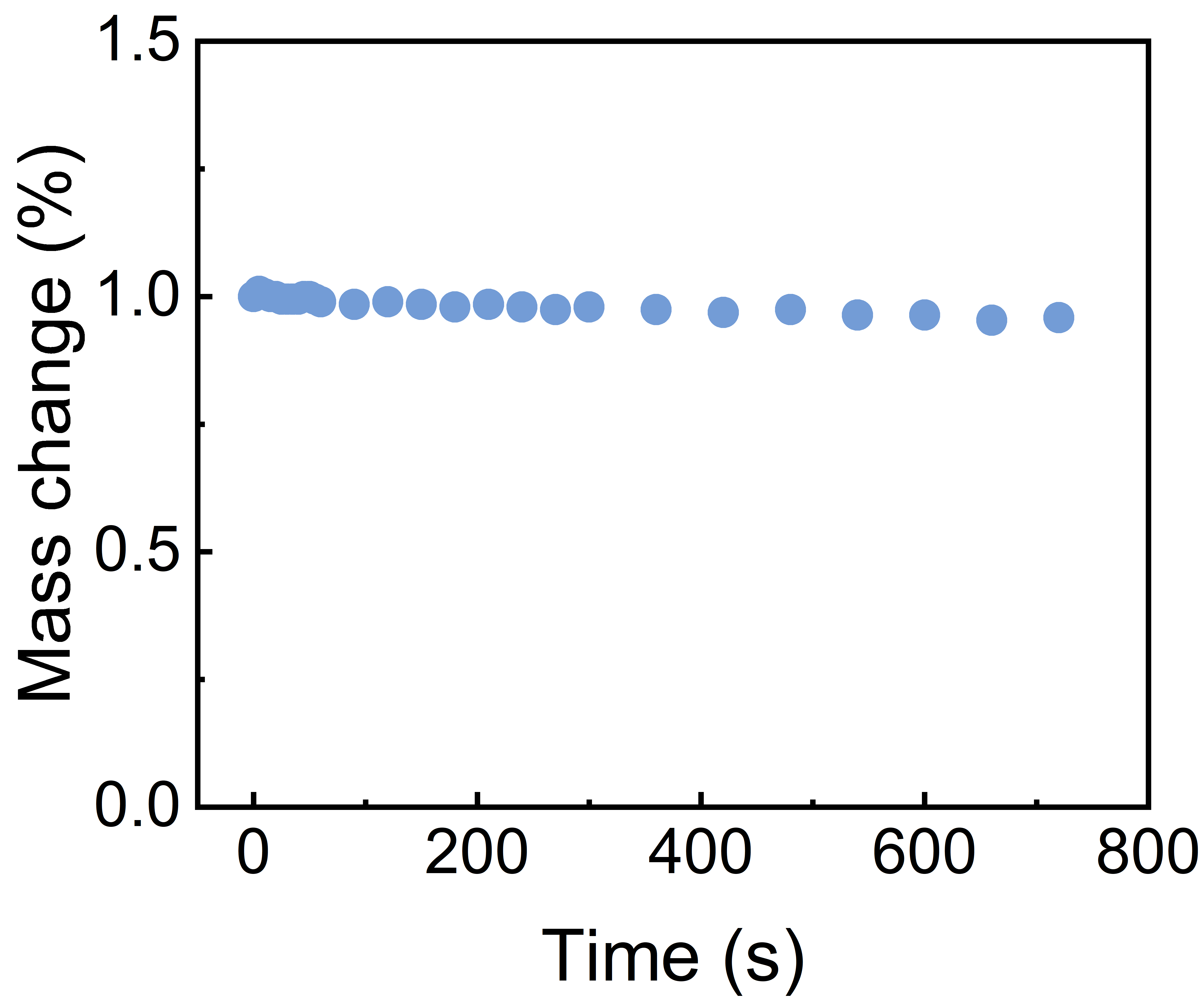


**Fig. S15** The long-term mass change of PVDF-HFP-PEO gel in an environment of 50 ℃

*
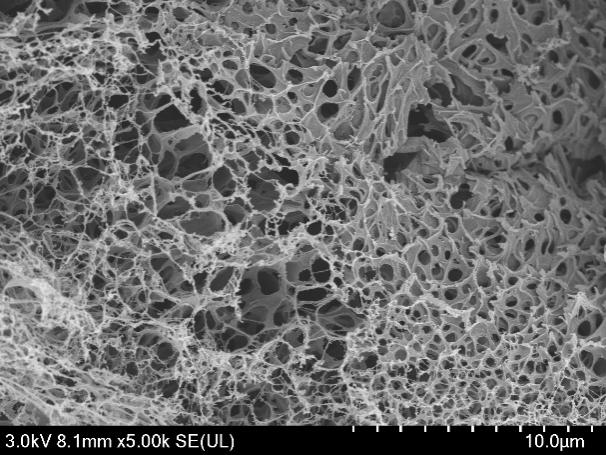

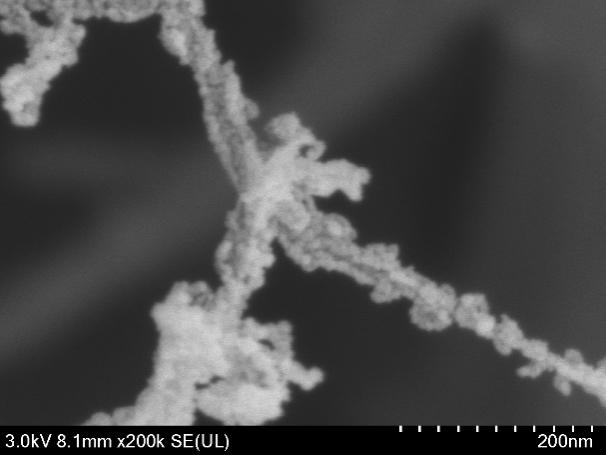
*

**Fig. S16** SEM images of the surface of the carbonized PI film by laser sintering method

*
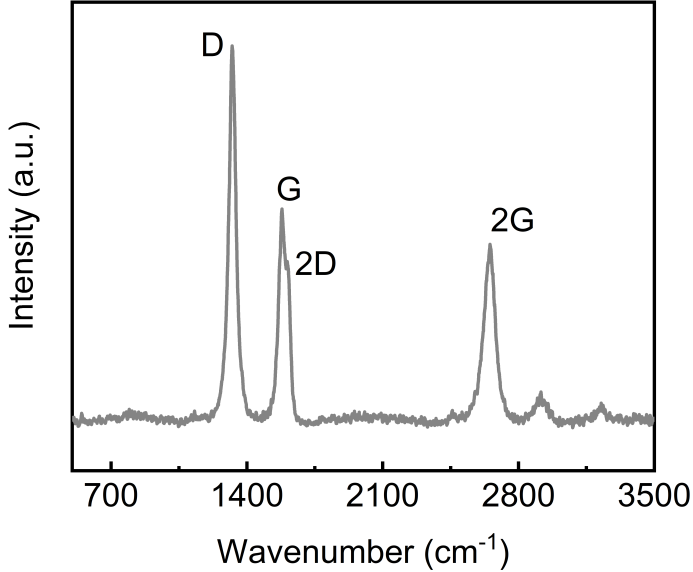
*

**Fig. S17** Raman spectrum of C@PI film


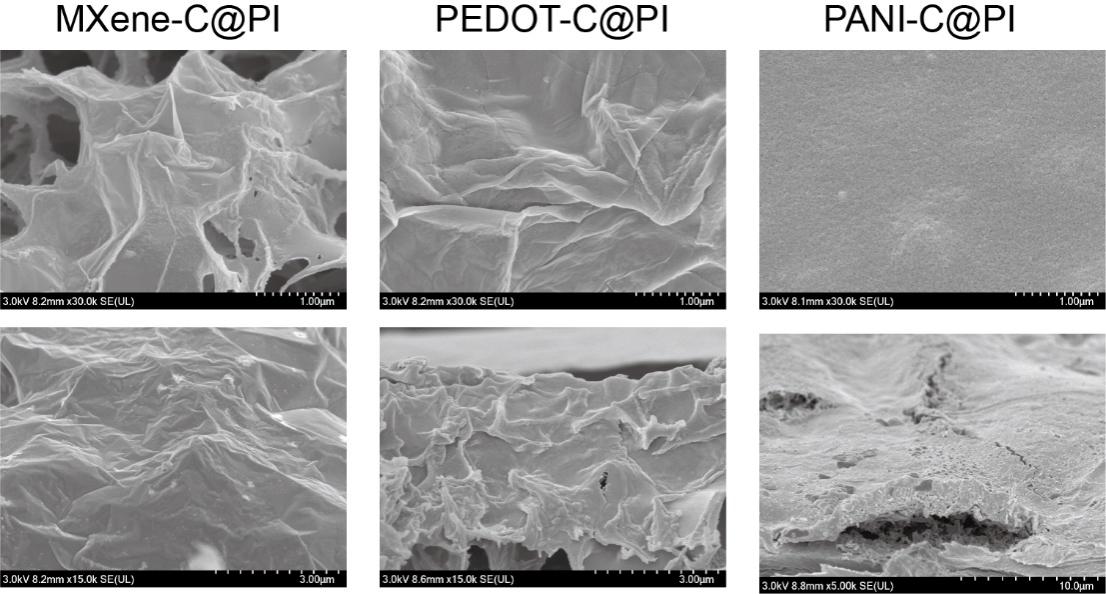


**Fig. S18** SEM images of the surface and section of MXene-C@PI, PEDOT-C@PI and PANI-C@PI films


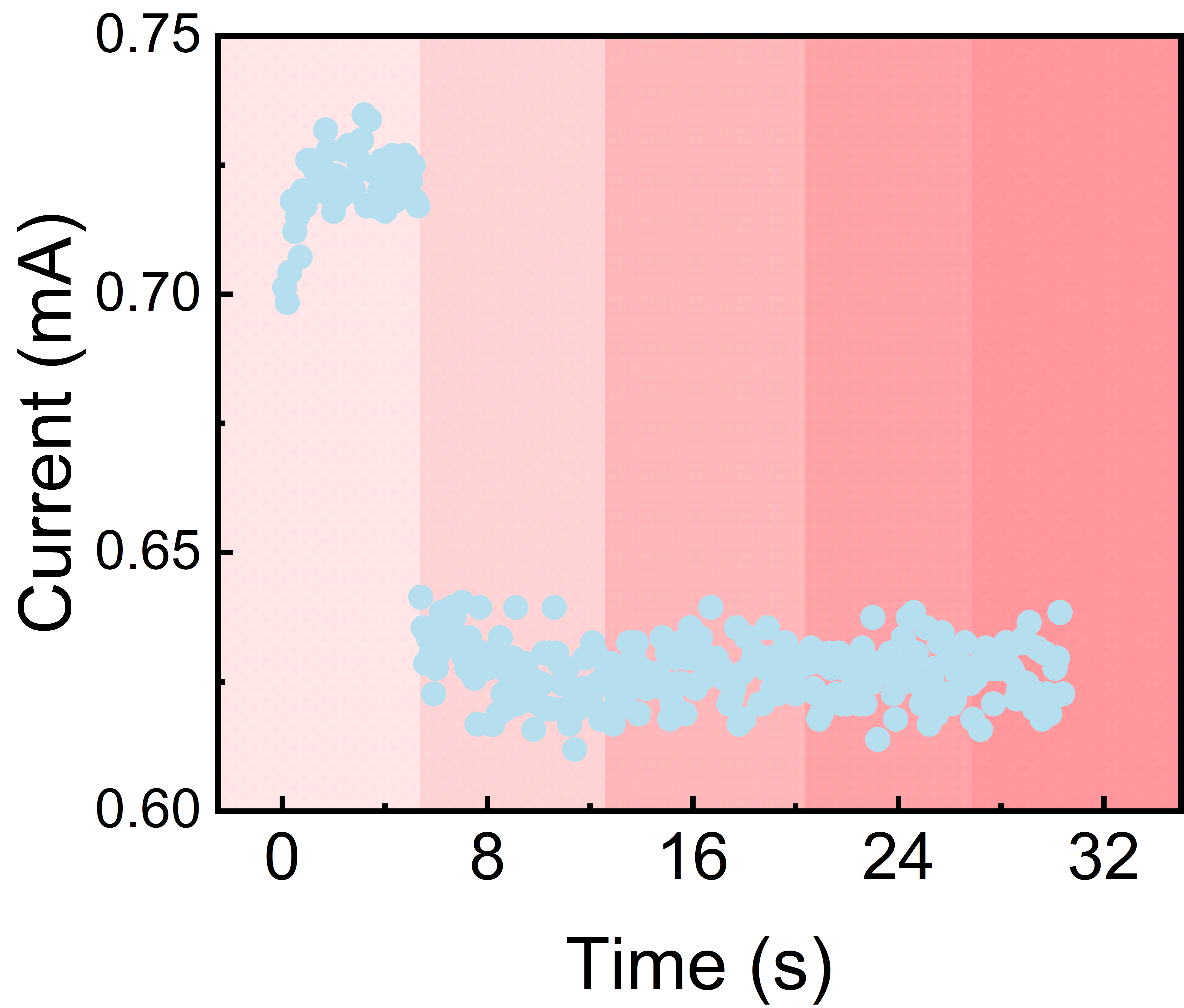


**Fig. S19** The I-t curve of the PANI-C@PI electrode film under applying different bending angles (0°, 10°, 20°, 30°, 40°)


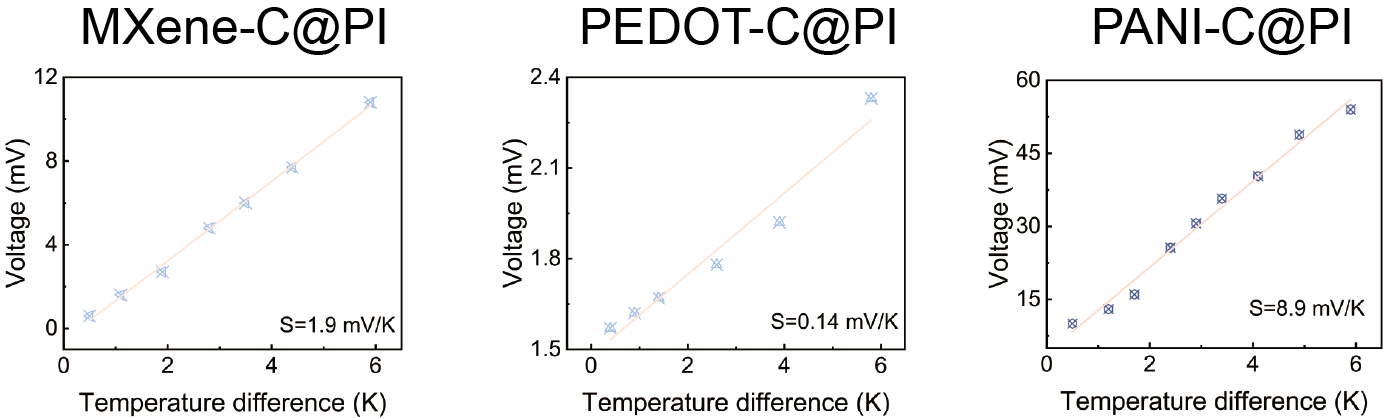


**Fig. S20** The output voltages of the i-TE devices assembled by MXene-C@PI, PEDOT-C@PI, and PANI-C@PI films under different temperature differences


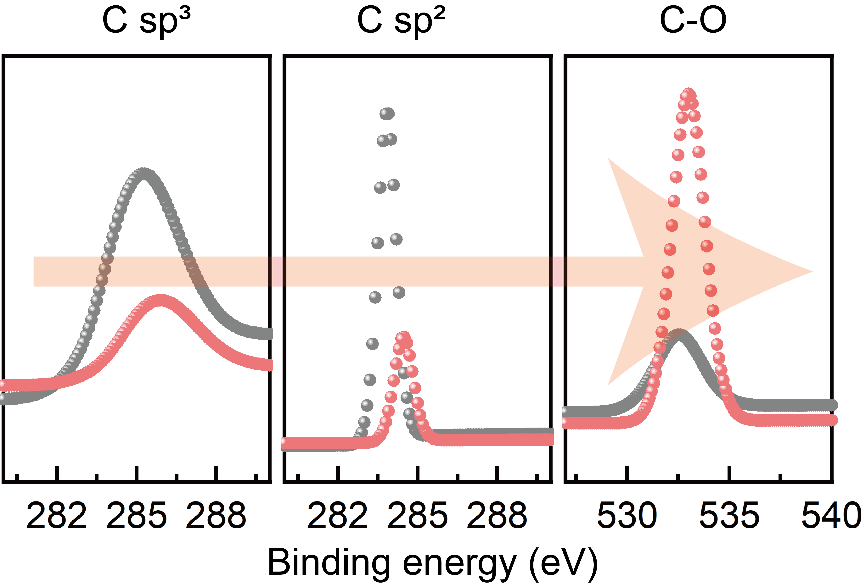


**Fig. S21** The element convolution XPS spectra of C@PI film (black). The red plots represents the C@PI film after being soaked with electrolyte. An obvious red shift can be found after being soaked with Li electrolyte, which can demonstrate that the binding force of the chemical bond is weakened due to coordination after ions are adsorbed on the surface of the material


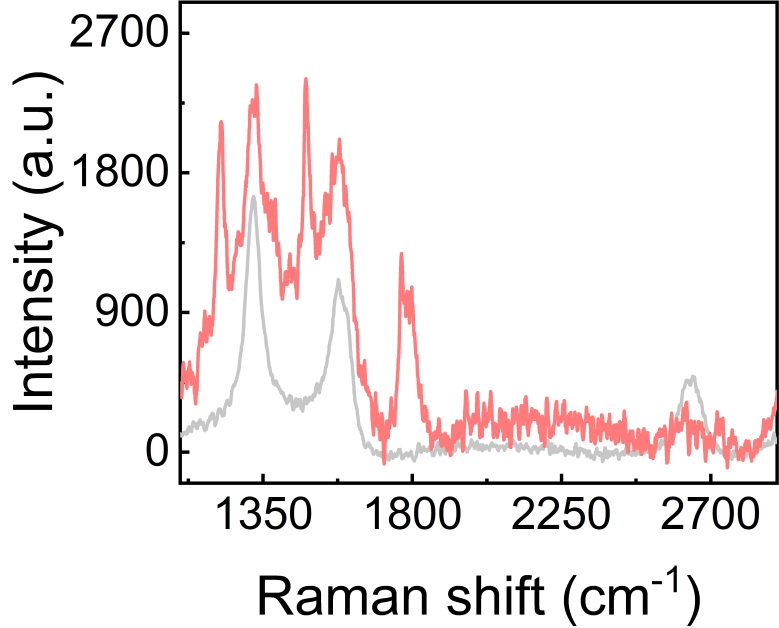


**Fig. S22** Raman spectrum of MXene-C@PI film


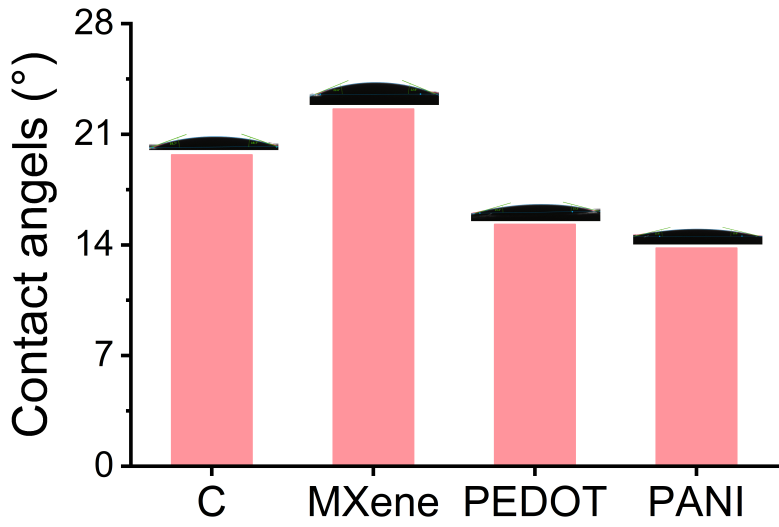


**Fig. S23** The contact angles of the four electrodes to the electrolyte


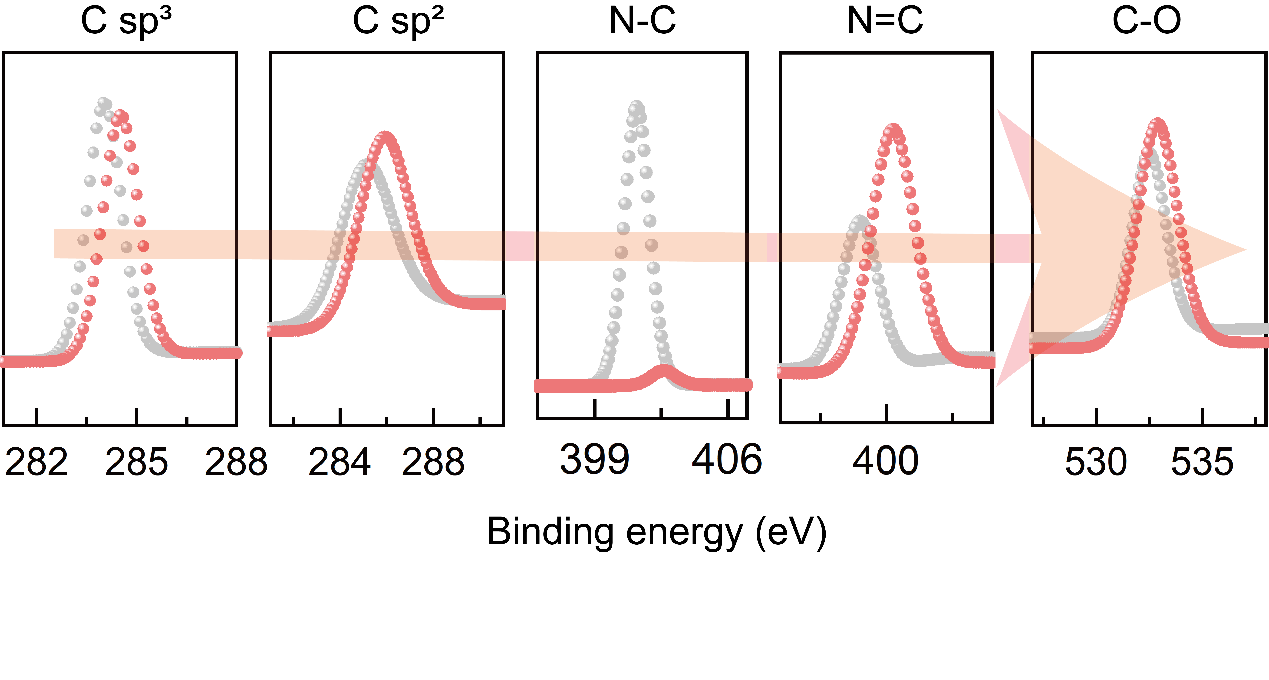


**Fig. S24** The element convolution XPS spectra of PANI-C@PI film (black). The red plots represents the element convolution XPS spectra of PANI-C@PI film after being soaked with electrolytes


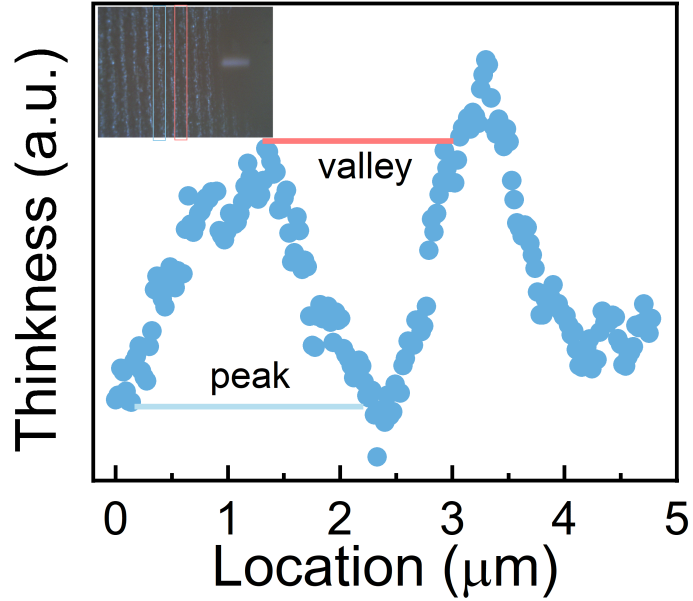


**Fig. S25** AFM images of PANI-C@PI film, which shows the undulating mountain structure


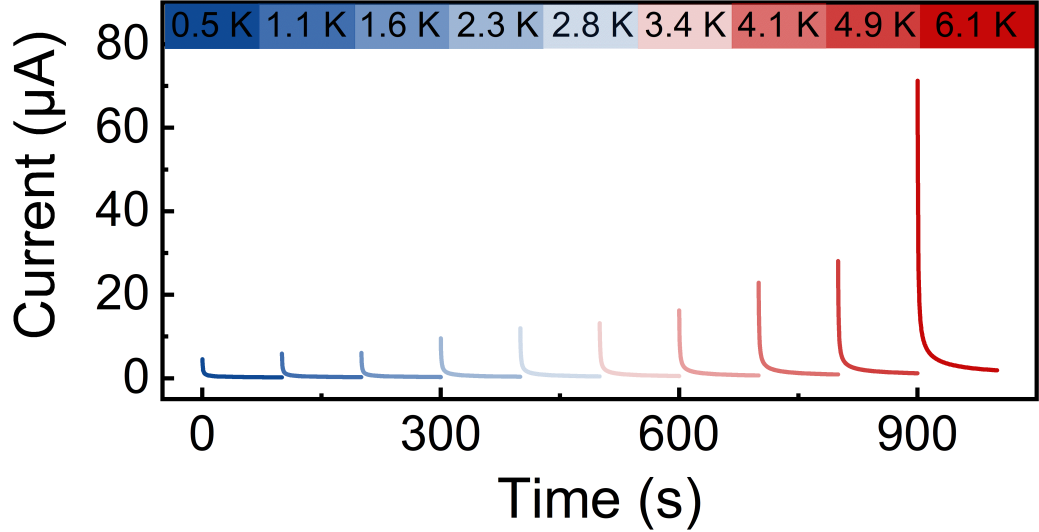


**Fig. S26** The output current of the i-TE devices assembled by PANI-C@PI film under different temperature differences


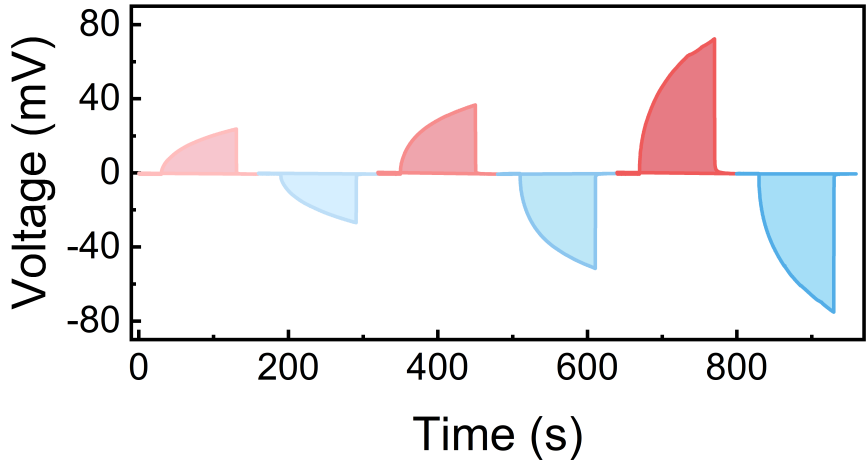


**Fig. S27** The voltages curve obtained by applying several temperature differences in the opposite direction to the device


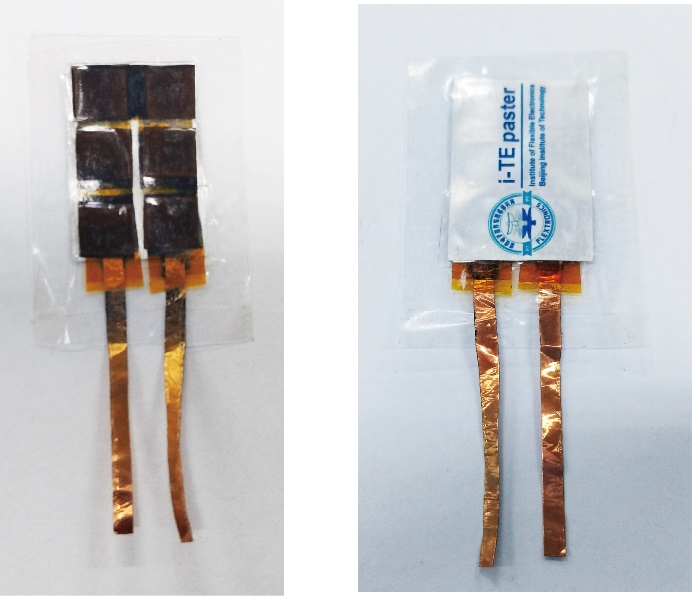


**Fig. S28** The optical photographs of the TCSC supported by i-TE paster


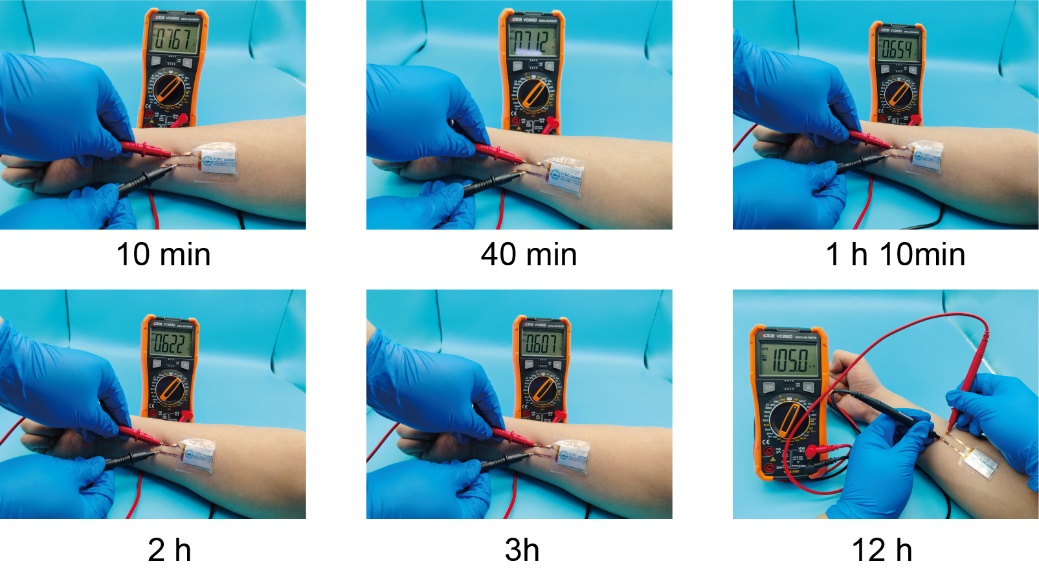


**Fig. S29** The thermal-voltage generated by i-TE paster by using a multimeter measuring at different times


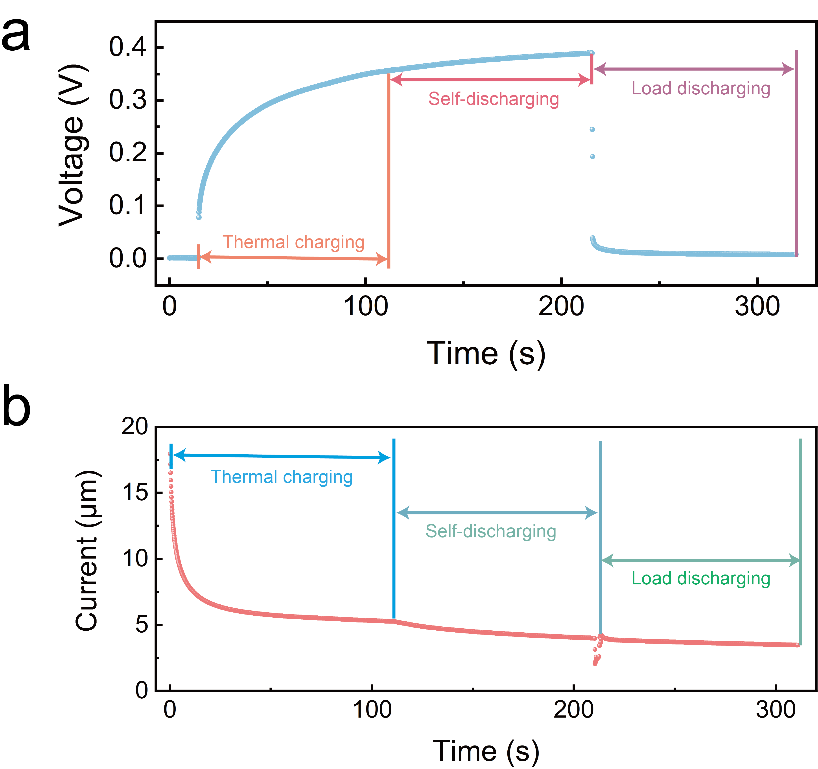


**Fig. S30** The V-t (**a**) and I-t (**b**) curves of the TCSC in the process of thermal charging, self-discharging and load discharging


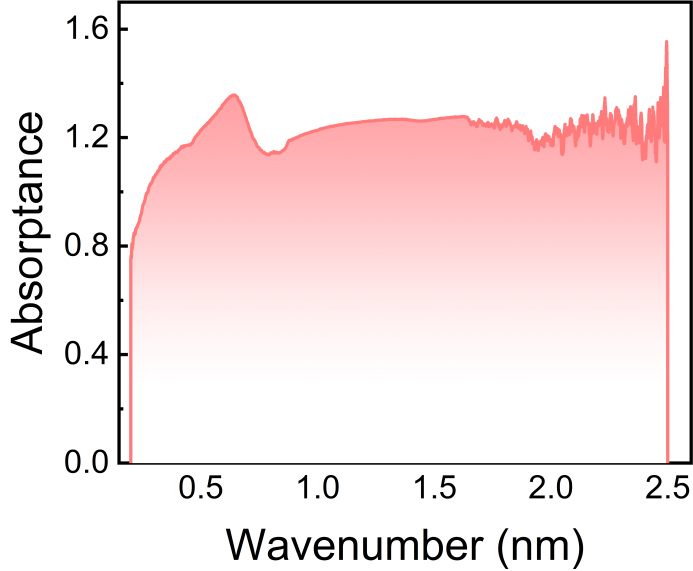


**Fig. S31** Solar absorbance of the PANI-C@PI film under different wavelengths of radiation

**Table S1** Comperison of Seebeck coefficients and ZT figure of gel-based i-TE materials

| No. | Materials | Seebeck coefficient (mV/K) | ZT figure | References |
| --- | --- | --- | --- | --- |
| 1 | PAAm/Fe-Alg gel | 1.43 | 0.002~0.003 | [S19] |
| 2 | PAAm-FeCN gel | 1.37 | 0.001 | [S20] |
| 3 | PAAm-Fe gel | -1.65 | 0.002 | [S20] |
| 4 | LCI12-7 gel | -5.9 | 3.8×10^-7^ | [S21] |
| 5 | WPU-ChCl-EG gel | 19.5 | 0.22 | [S22] |
| 6 | DMPAM NPs gel | 6.1 | 7.2×10^–4^ | [S23] |
| 7 | AAc NPs gel | -6.7 | 8.8×10^–4^ | [S23] |
| 8 | Ion organic gel | 21.8 | 0.35 | This work |

**Table S2** Comperison of Seebeck coefficients (equivalent sensitivity) of i-TE devices.

| No. | Devices | Seebeck coefficient (mV/K) | References |
| --- | --- | --- | --- |
| 1 | MIR | 4.1 | [S24] |
| 2 | SHR-E | 1.57 | [S25] |
| 3 | CPN noncontact temperature sensor | 0.0305 | [S26] |
| 4 | TCSC | 8 | [S27] |
| 5 | ITE(S)C | 18.55 | [S28] |
| 6 | SPTC | 6.5 | [S29] |
| 7 | Te-based sensor | 0.941 | [S30] |
| 8 | CTCC | 25.2 | [S31] |
| 9 | TCHS | 12.1 | [S32] |
| 10 | PANI-E skin | 0.11 | [S33] |
| 11 | MCP sensor | -5×10^–3^ | [S34] |
| 12 | i-TE paster | 28 | This work |

**Supplementary References**

1. R. Roy, K.C. Stevens, K.A. Treaster, B.S. Sumerlin, A.J.H. McGaughey et al., Intrinsically thermally conductive polymers. Mater. Horiz. **11**(14), 3267–3286 (2024). <https://doi.org/10.1039/d3mh01796f>
2. W. Zhao, R. Hu, Toward high-thermal-conductivity polymers. Matter **4**(12), 3799–3801 (2021). <https://doi.org/10.1016/j.matt.2021.10.029>
3. S. Xu, S. Cai, Z. Liu, Thermal conductivity of polyacrylamide hydrogels at the nanoscale. ACS Appl. Mater. Interfaces **10**, 36352 (2018). <https://doi.org/10.1021/acsami.8b09891>
4. X.-F. Zhang, X. Ma, T. Hou, K. Guo, J. Yin et al., Inorganic salts induce thermally reversible and anti-freezing cellulose hydrogels. Angew. Chem. Int. Ed. **58**(22), 7366–7370 (2019). <https://doi.org/10.1002/anie.201902578>
5. S. Bhattacharya, Crystal packing driven comparable thermal expansion of polymorphs of a tripodal molecule and guest-dependent expansion of its isostructural solvates. Cryst. Growth Des. **21**(2), 1290–1296 (2021). <https://doi.org/10.1021/acs.cgd.0c01559>
6. B. Garai, V. Bon, A. Efimova, M. Gerlach, I. Senkovska et al., Reversible switching between positive and negative thermal expansion in a metal–organic framework DUT-49. J. Mater. Chem. A **8**(39), 20420–20428 (2020). <https://doi.org/10.1039/D0TA06830F>
7. L. Deschênes, J. Lyklema, C. Danis, F. Saint-Germain, Phase transitions in polymer monolayers: application of the Clapeyron equation to PEO in PPO-PEO Langmuir films. Adv. Colloid Interface Sci. **222**, 199–214 (2015). <https://doi.org/10.1016/j.cis.2014.11.002>
8. N. Ding, S.W. Chien, T.L.D. Tam, X. Li, G. Wu et al., Revealing phase transitions in poly(ethylene oxide)-based electrolyte for room-temperature solid-state batteries. Adv. Energy Mater. **14**(40), 2402986 (2024). <https://doi.org/10.1002/aenm.202402986>
9. A.A. Eddib, D.D.L. Chung, Electric permittivity of carbon fiber. Carbon **143**, 475–480 (2019). <https://doi.org/10.1016/j.carbon.2018.11.028>
10. D. Thuau, K. Kallitsis, S. Ha, F. Bargain, T. Soulestin et al., High and temperature-independent dielectric constant dielectrics from PVDF-based terpolymer and copolymer blends. Adv. Electron. Mater. **6**, 1901250 (2020). <https://doi.org/10.1002/aelm.201901250>
11. T. Zheng, J. Xiong, X. Shi, B. Zhu, Y.-J. Cheng et al., Cocktail therapy towards high temperature/high voltage lithium metal battery *via* solvation sheath structure tuning. Energy Storage Mater. **38**, 599–608 (2021). <https://doi.org/10.1016/j.ensm.2021.04.002>
12. Z. Wang, Y. Sun, Y. Mao, F. Zhang, L. Zheng et al., Highly concentrated dual-anion electrolyte for non-flammable high-voltage Li-metal batteries. Energy Storage Mater. **30**, 228 (2020). <https://doi.org/10.1016/j.ensm.2020.05.020>
13. T. Zheng, J. Xiong, B. Zhu, X. Shi, Y.-J. Cheng et al., From–20 ℃ to 150 ℃: a lithium secondary battery with a wide temperature window obtained *via* manipulated competitive decomposition in electrolyte solution. J. Mater. Chem. A **9**(14), 9307–9318 (2021).
14. X. Zhang, X. Chen, X. Cheng, B. Li, X. Shen et al., From -20 °C to 150 °C: a lithium secondary battery with a wide temperature window obtained via manipulated competitive decomposition in electrolyte solution. Angew. Chem. Int. Ed. **57**, 5301 (2018). <https://doi.org/10.1002/anie.201801513>
15. A. Su, P. Guo, J. Li, D. Kan, Q. Pang et al., An organic–inorganic semi-interpenetrating network ionogel electrolyte for high-voltage lithium metal batteries. J. Mater. Chem. A **8**(9), 4775–4783 (2020). <https://doi.org/10.1039/c9ta05804d>
16. S. Muench, R. Burges, A. Lex-Balducci, J.C. Brendel, M. Jäger et al., Printable ionic liquid-based gel polymer electrolytes for solid state all-organic batteries. Energy Storage Mater. **25**, 750–755 (2020). <https://doi.org/10.1016/j.ensm.2019.09.011>
17. S. Muench, R. Burges, A. Lex-Balducci, J.C. Brendel, M. Jäger et al., Printable ionic liquid-based gel polymer electrolytes for solid state all-organic batteries. Energy Storage Mater. **25**, 750–755 (2020). <https://doi.org/10.1016/j.ensm.2019.09.011>
18. H. Wang, S.C. Kim, T. Rojas, Y. Zhu, Y. Li et al., Correlating Li-ion solvation structures and electrode potential temperature coefficients. J. Am. Chem. Soc. **143**(5), 2264–2271 (2021). <https://doi.org/10.1021/jacs.0c10587>
19. P. Peng, J. Zhou, L. Liang, X. Huang, H. Lv et al., Regulating thermogalvanic effect and mechanical robustness *via* redox ions for flexible quasi-solid-state thermocells. Nano-Micro Lett. **14**(1), 81 (2022). <https://doi.org/10.1007/s40820-022-00824-6>
20. C. Xu, Y. Sun, J. Zhang, W. Xu, H. Tian, Adaptable and wearable thermocell based on stretchable hydrogel for body heat harvesting. Adv. Energy Mater. **12**(42), 2201542 (2022). <https://doi.org/10.1002/aenm.202201542>
21. S. Park, B. Kim, C. Cho, E. Kim, Mesogenic polymer composites for temperature-programmable thermoelectric ionogels. J. Mater. Chem. A **10**(26), 13958–13968 (2022). <https://doi.org/10.1039/d2ta03120e>
22. Y. Zhao, H. Cheng, Y. Li, J. Rao, S. Yue et al., Quasi-solid conductive gels with high thermoelectric properties and high mechanical stretchability consisting of a low cost and green deep eutectic solvent. J. Mater. Chem. A **10**(8), 4222–4229 (2022). <https://doi.org/10.1039/D1TA09707E>
23. B. Guo, Y. Hoshino, F. Gao, K. Hayashi, Y. Miura et al., Thermocells driven by phase transition of hydrogel nanoparticles. J. Am. Chem. Soc. **142**(41), 17318–17322 (2020). <https://doi.org/10.1021/jacs.0c08600>
24. Y. Li, W. Wang, X. Cui, N. Li, X. Ma et al., Self-powered machine-learning-assisted material identification enabled by a thermogalvanic dual-network hydrogel with a high thermopower. Small **21**(1), e2405911 (2025). <https://doi.org/10.1002/smll.202405911>
25. X. Luo, C. Chen, Z. He, M. Wang, K. Pan et al., A bionic self-driven retinomorphic eye with ionogel photosynaptic retina. Nat. Commun. **15**(1), 3086 (2024). <https://doi.org/10.1038/s41467-024-47374-6>
26. F.L. Gao, P. Min, Q. Ma, T. Zhang, Z.Z. Yu et al., Multifunctional thermoelectric temperature sensor for noncontact information transfer and tactile sensing in human-machine interaction. Adv. Funct. Mater. **34**(1), 2309553 (2024). <https://doi.org/10.1002/adfm.202309553>
27. K.S. L, L.H. T, C. Yu, Thermally chargeable solid-state supercapacitor. Adv. Energy Mater. **6**(18), 1600546 (2016). <https://doi.org/10.1002/aenm.201600546>
28. Y.T. Malik, Z.A. Akbar, J.Y. Seo, S. Cho, S.Y. Jang et al., Self-healable organic–inorganic hybrid thermoelectric materials with excellent ionic thermoelectric properties. Adv. Energy Mater. **12**(6), 2103070 (2022). <https://doi.org/10.1002/aenm.202103070>
29. L. Liu, D. Zhang, P. Bai, Y. Mao, Q. Li et al., Strong tough thermogalvanic hydrogel thermocell with extraordinarily high thermoelectric performance. Adv. Mater. **35**(32), e2300696 (2023). <https://doi.org/10.1002/adma.202300696>
30. L. Li, S. Zhao, W. Ran, Z. Li, Y. Yan, B. Zhong, Z. Lou, L. Wang, and G. Shen,Dual sensing signal decoupling based on tellurium anisotropy for VR interaction and neuro-reflex system application. Nat. Commun. **13**, 5975 (2022). <https://doi.org/10.1038/s41467-022-33716-9>
31. Z. Hu, S. Chang, C. Cheng, C. Sun, J. Liu et al., Dual sensing signal decoupling based on tellurium anisotropy for VR interaction and neuro-reflex system application. Energy Storage Mater. **58**, 353 (2023). <https://doi.org/10.1016/j.ensm.2023.03.037>
32. T. Meng, Y. Xuan, X. Zhang, A thermally chargeable hybrid supercapacitor with high power density for directly converting heat to electricity. ACS Appl. Energy Mater. **4**(6), 6055–6061 (2021). <https://doi.org/10.1021/acsaem.1c00905>
33. P.C. Zhu, Y.L. Wang, Y. Wang, H.Y. Mao, Q. Zhang et al., Flexible 3D architectured piezo/thermoelectric bimodal tactile sensor array for E-skin application. Adv. Energy Mater. **10**(39), 2001945 (2020). <https://doi.org/10.1002/aenm.202001945>
34. F.-L. Gao, J. Liu, X.-P. Li, Q. Ma, T. Zhang et al., Ti_3_C_2_T*_x_* MXene-based multifunctional tactile sensors for precisely detecting and distinguishing temperature and pressure stimuli. ACS Nano **17**(16), 16036–16047 (2023). <https://doi.org/10.1021/acsnano.3c04650>
